# Multi-Walled Carbon Nanotubes Decorated with Guanidinylated Dendritic Molecular Transporters: An Efficient Platform for the Selective Anticancer Activity of Doxorubicin

**DOI:** 10.3390/pharmaceutics13060858

**Published:** 2021-06-09

**Authors:** Kyriaki-Marina Lyra, Archontia Kaminari, Katerina N. Panagiotaki, Konstantinos Spyrou, Sergios Papageorgiou, Elias Sakellis, Fotios K. Katsaros, Zili Sideratou

**Affiliations:** 1 Institute of Nanoscience and Nanotechnology, National Center for Scientific Reasearch ‘‘Demokritos”, 15310 Aghia Paraskevi, Greece; k.lyra@inn.demokritos.gr (K.-M.L.); a.kaminari@inn.demokritos.gr (A.K.); k.panagiotaki@inn.demokritos.gr (K.N.P.); s.papageorgiou@inn.demokritos.gr (S.P.); e.sakellis@inn.demokritos.gr (E.S.); f.katsaros@inn.demokritos.gr (F.K.K.); 2 Department of Material Science & Engineering, University of Ioannina, 45110 Ioannina, Greece; konstantinos.spyrou1@gmail.com

**Keywords:** drug delivery system, cancer cell specificity, doxorubicin, hyperbranched polyethyleneimine, carbon nanotubes, guanidinium, molecular transporters

## Abstract

An efficient doxorubicin (DOX) drug delivery system with specificity against tumor cells was developed, based on multi-walled carbon nanotubes (MWCNTs) functionalized with guanidinylated dendritic molecular transporters. Acid-treated MWCNTs (oxCNTs) interacted both electrostatically and through hydrogen bonding and van der Waals attraction forces with guanidinylated derivatives of 5000 and 25,000 Da molecular weight hyperbranched polyethyleneimine (GPEI5K and GPEI25K). Chemical characterization of these GPEI-functionalized oxCNTs revealed successful decoration with GPEIs all over the oxCNTs sidewalls, which, due to the presence of guanidinium groups, gave them aqueous compatibility and, thus, exceptional colloidal stability. These GPEI-functionalized CNTs were subsequently loaded with DOX for selective anticancer activity, yielding systems of high DOX loading, up to 99.5% encapsulation efficiency, while the DOX-loaded systems exhibited pH-triggered release and higher therapeutic efficacy compared to that of free DOX. Most importantly, the oxCNTs@GPEI5K-DOX system caused high and selective toxicity against cancer cells in a non-apoptotic, fast and catastrophic manner that cancer cells cannot recover from. Therefore, the oxCNTs@GPEI5K nanocarrier was found to be a potent and efficient nanoscale DOX delivery system, exhibiting high selectivity against cancerous cells, thus constituting a promising candidate for cancer therapy.

## 1. Introduction

In recent years, chemotherapy has been the most common strategy for cancer treatment. Chemotherapy drugs act in different ways to kill or to inhibit the uncontrolled growth and proliferation of cancer cells. Most of them attack the cells’ DNA, avoiding mitosis (cell division) or inducing DNA damage. However, due to their nonspecific distribution and lack of selectivity, these drugs cannot distinguish cancer cells from normal cells, causing severe toxic side effects [1,2]. Additionally, although initially chemotherapy can be effective against many types of cancer, over time cancers have the ability to develop resistance to these drugs or other mechanisms, such as DNA mutations and metabolic alterations, inhibiting their actions or causing their degradation. Thus, various advanced drug delivery systems (DDSs) based on different nanoscale materials have been investigated for cancer therapy, due to their ability to carry bioactive molecules and their potential to target cancer tumors [3,4,5,6,7]. In particular, these DDSs can accumulate at the tumor site either through the enhanced permeability and retention effect (EPR) or through their functionalization with suitable targeting moieties. This would, at least in principle, result in a decrease in the associated side effects and an increase in treatment efficiency [8]. So far, numerous nanoscale materials, including inorganic nanoparticles [9,10], liposomes [11,12], polymeric nanoparticles [12,13,14] and carbon-based materials [15,16], have been proposed as drug nanocarriers; however, only a handful have been approved, due to concerns and challenges on biocompatibility, pharmacokinetics and in vivo targeting efficacy [17]. Out of the various DDSs currently under investigation for cancer therapy, multi-walled carbon nanotubes (MWCNTs) are considered to have great potential due to their unique structural characteristics, sufficient surface-to-volume ratio as well as their excellent mechanical, electrical, thermal, optical and chemical properties [18,19]. Modification of their surfaces with appropriate functional groups allows optimization of their properties. Moreover, various anticancer drugs have been loaded on the surface of carbon nanotubes (CNTs) through covalent or non-covalent bonding and efficiently delivered into cancer cells, enhancing their anticancer activity [20].

However, the application of DDSs based on MWNTs faces critical challenges due to their potential toxicity and extremely hydrophobic nature. It is known that MWCNTs exhibit low aqueous dispersibility due to their tendency to form aggregates. Thus, surface functionalization of MWCNTs following various covalent and non-covalent processes has been proposed. Decoration with oxygen-containing groups [21] can render them water-soluble, while functionalization with polymers [22,23,24] or surfactants [25,26] has been reported to improve not only their aqueous dispersibility, but also their biocompatibility and cellular uptake [27].

Dendritic polymers are a class of highly branched macromolecules of nanosized dimensions, consisting of dendrons, dendrimers and hyperbranched polymers. Hyperbranched polymers, such as hyperbranched polyethyleneimine, have a similar structure to dendrimers, consisting of repeating units and surface end groups [28,29]. These end groups can be easily functionalized or multi-functionalized, affording a variety of nanomaterials with tailor-made properties able to be used in biomedical or other applications. Additionally, a great number of these terminal groups enhances their binding to cells due to the so-called multivalent effect [30], as they may allow the dendritic polymer to access several cellular receptors simultaneously. Combining these properties with their ability to encapsulate bioactive molecules in their interior, dendritic polymers have been extensively investigated as effective drug delivery systems [31,32,33,34].

Guanidinylated dendrimers have been investigated as molecular transporters, which can promote the transport of bioactive compounds across biological membranes [35,36,37,38]. These functionalized dendrimers exhibited analogous behavior with the most known molecular transporters, i.e., the so-called cell-penetrating peptides, the structure of which is characterized by an appropriate array of guanidinium groups [39,40]. In our previous work, poly(propylene imine) dendrimers of the third and fourth generation were functionalized, introducing guanidinium groups at various substitution degrees either directly to the polymeric scaffold or though alkyl spacers. It was proven that these guanidinylated dendrimers can be used as transporting agents for drug delivery since they can efficiently penetrate through cellular membranes, internalize into cells and specifically localize in subcellular organelles and the cytosol [41]. Additionally, it has been shown that at the first stage of the transport process to the cell interior, guanidinium groups interact both electrostatically and through hydrogen bonding with phosphate, carboxylate or sulfate groups located on the cell surface due to electronic and geometrical complementarity. In this context, it has also been shown that strong binding of guanidinium to the phosphate group, for instance, is amplified by multivalent effects [30]. Thus, multivalent effects act synergistically with hydrogen and electrostatic bonding, enhancing the binding of recognizable groups located in close proximity to the dendrimer, with the complementary acidic groups situated on the cell surface. The neutralization of charges as well as the presence of a transmembrane potential, due to the potassium ion concentration gradient, are the prerequisite forces for cellular entry.

Doxorubicin (DOX) is a known anticancer drug, which acts as a DNA intercalator and damaging agent, widely used for treatment against various types of cancer [42]. Due to its side effects such as systemic toxicity [43,44] and chemo-resistance [45], its application is limited [46]. To overcome these obstacles, several nanoparticulate systems, including dendritic polymers [31,47,48] and carbon nanotubes [49,50,51], have been proposed for DOX delivery. For example, Liao et al. [52] prepared a series of PEGylated G5 PAMAM dendrimers with different PEGylation degrees in order to be used as efficient DOX delivery systems. It was found that the encapsulated DOX exhibited improved antitumor activity compared to free DOX and successful internalization into the cell nucleus, analogous to the free drug. In another work, a nanocarrier based on hyperbranched polyethyleneimine functionalized with triphenylphosphonium groups was developed [53]. DOX was efficiently encapsulated into this nanocarrier and delivered to mitochondria of prostate cancer cells, resulting in enhancement of its anticancer activity at extremely low doses. On the other hand, taking advantage of the aromatic structure of DOX and its ability to establish non-covalent interactions with the sidewalls of CNTs, Wang et al. [54] evaluated the adsorption and desorption capacity of DOX on oxidized MWCNTs (oxCNTs). It was found that DOX can effectively be loaded to oxCNTs due to the π-π stacking interactions between the quinine portion of DOX and the graphitic sidewalls of oxCNTs as well as the hydrogen bonds and/or electrostatic forces between the amino and hydroxyl groups of DOX with oxygen-containing groups of oxCNTs. Moreover, it was shown that DOX can be released more promptly at a pH of 5.5 compared to 7.4, suggesting that this system can be used for efficient targeted delivery to cancer cells, as it is known that the tumor microenvironment tends to be more acidic than that of normal cells [55]. In another similar work, DOX was attached to the surface of MWCNTs dispersed in aqueous solution of Pluronic F127 though non-covalent bonding, and subsequently the anticancer activity of this system against human breast cancer cells MCF-7 was assessed. The administration of loaded DOX led to enhanced cell proliferation inhibition compared to free DOX.

Recently, functional dendritic polymers have been used to covalently modify the surface of MWCNTs, affording hybrid materials to be used as drug delivery systems [22,56,57]. In this context, oxidized MWCNTs were covalently modified with amino-terminated PAMAM dendrimers multi-functionalized with fluorescein and folate moieties, and then DOX was attached to this system though π-π stacking interactions [58]. Up to 97.8% DOX loading and encapsulation efficiency was achieved, while the DOX-loaded system showed pH-responsive release properties, where DOX readily released under acidic environments in contrast to physiological pH conditions. Moreover, it was shown that this system was able to specifically deliver DOX to cancer cells overexpressing folate receptors and demonstrated improved anticancer activity compared to the free drug. Analogous results were obtained in another similar work for DOX delivery, presented by the same group, where, this time, oxidized MWCNTs were covalently functionalized with hyperbranched polyethyleneimine, bearing again fluorescein and folate moieties [59]. Hyperbranched polyethyleneimine (PEI) is a water-soluble dendritic polymer containing tertiary, secondary and primary amino groups, which can easily interact with the oxygen-containing groups located on the surface of oxCNTs, leading to the formation of grafted CNTs with amino-functionalized dendritic structure [50,60].

In the present work, for the first time, we functionalized MWCNTs with guanidinylated dendritic molecular transporters through non-covalent interactions to be used as efficient doxorubicin delivery systems with enhanced cell-penetrating capability and specific toxicity against cancer cells. Specifically, guanidinylated derivatives of hyperbranched polyethyleneimine with molecular weights 5000 and 25,000 Da (GPEI5K and GPEI25K), having analogous chemical structure with guanidinylated poly(propylene imine) dendrimers, were synthesized in order to be used as transporting agents for drug delivery. Subsequently, these guanidinylated derivatives were non-covalently attached onto oxidized multi-walled carbon nanotubes (oxCNTs), affording novel water-soluble hybrid materials (oxCNTs@GPEI5K and oxCNTs@GPEI25K). Thus, these guanidinylated PEI derivatives interacted electrostatically and also through hydrogen bonding and van der Waals attraction forces with oxCNTs. The as-prepared functionalized CNTs were characterized by a variety of physicochemical techniques (FTIR, Raman, SEM, TEM, AFM, etc.), while their excellent aqueous dispersibility was evaluated employing UV–vis spectroscopy and *ζ*-potential measurements. Ultimately, the guanidinylated PEI-functionalized MWCNTs were used as DOX-loaded nanocarriers. After the evaluation of the DOX loading capacity of oxCNTs@GPEIs and the pH-triggered DOX release, the cell-penetrating capability as well as the targeting efficacy of the obtained DOX-loaded systems to tumor cells was studied by intracellular uptake through flow cytometry and confocal microscopy analysis on DU145 human prostate cancer cells and on the HEK293 normal human kidney cell line. Additionally, the in vitro anticancer activity of DOX-loaded systems was investigated in terms of their cytotoxicity on aggressive DOX-resistant DU145 and PC3 human prostate carcinoma cell lines as well as on normal HEK293 cells, while their effect on cell apoptosis/necrosis was studied employing flow cytometry. The results obtained from this study may provide a basis for the rational design of a drug delivery systems based on CNTs functionalized with dendritic molecular transporters for various therapeutic applications, including cancer therapy.

## 2. Materials and Methods

### 2.1. Chemicals and Reagents

Multi-walled carbon nanotubes containing carboxyl groups > 8% *w*/*w* (CNTs), hyperbranched polyethyleneimine (PEI) of 5000 Da (Lupasol**^®^** G100, water-free, 99%) and 25,000 Da molecular weight (Lupasol**^®^** WF, water-free, 99%) and doxorubicin hydrochloride were kindly donated by Glonatech S.A (Athens, Greece), BASF (Ludwigshafen, Germany) and Regulon S.A. (Athens, Greece), respectively. 1*H*-Pyrazole-1-carboxamidine hydrochloride, *N*,*N*-diisopropylethylamine (DIPEA), thiazolyl blue tetrazolium bromide (MTT), rhodamine B isothiocyanate and dialysis tubes (M.W. cut-off: 1200) were purchased from Sigma-Aldrich Ltd. (Poole, UK). D-MEM low glucose with phenol red, fetal bovine serum (FBS), penicillin/streptomycin, L-glutamine, phosphate buffer saline (PBS) and trypsin/EDTA were purchased from Biochrom (Berlin, Germany). FITC Annexin V Apoptosis Detection Kit with 7-AAD was purchased from BioLegend Way (San Diego, CA, USA). High-purity solvents such as *N*,*N*-dimethylformamide (DMF), diethyl ether, methanol and isopropanol were obtained from Merck KGaA (Calbiochem**^®^**, Darmstadt, Germany).

### 2.2. Synthesis of Guanidinylated Hyperbranched Polyethyleneimines

For the synthesis of guanidinylated hyperbranched polyethyleneimine derivatives (GPEI5K and GPEI25K), commercially available hyperbranched polyethyleneimines of molecular weight 5000 Da (PEI5K) and 25,000 Da (PEI25K) were used. The ratio of primary to secondary to tertiary amines was calculated using inverse-gated ^13^C NMR [61] at 1.06:1.26:1.00 for ΡΕΙ5Κ and 1.00:1.18:1.00 for ΡΕΙ25Κ.

Guanidinylated derivatives of hyperbranched polyethyleneimine with a 100% substitution degree of the primary amino groups were synthesized by a method analogous to the one described in the literature [62,63]. In brief, 0.01 mmol of PEI5K or PEI25K dissolved in 10 mL DMF was added to a DMF solution (10 mL) containing 0.4 or 2 mmol 1*H*-Pyrazole-1-carboxamidine hydrochloride and 0.8 mmol or 4 mmol DIPEA, respectively. The reaction mixture was left for 24 h at room temperature under continuous stirring and inert atmosphere. Then, the solution was concentrated, and the crude products were collected after precipitation in diethyl ether. The final products, GPEI5K and GPEI25K, were received after dialysis against deionized water for two days and lyophilized. The chemical structure of guanidinylated derivatives was characterized by ^1^H, ^13^C NMR and FTIR spectroscopies. Specifically, the successful introduction of the guanidinium moieties at the primary amino groups of PEI and the degree of substitution were established by ^1^H and ^13^C NMR spectroscopy.

^1^HNMR: (500 MHz, D_2_O): *δ* (ppm) = 3.25 (m, NCH_2_CH_2_NH-G), 2.70–2.50 (m, CH_2_ of PEI scaffold).

^13^CNMR (125.1 MHz, D_2_O): *δ* (ppm) = 157.0 (CH_2_NHC(NH_2_)NH_2_^+^), 54.0–50.0 (C_3-3_, C_3-2_ and C_3-1_), 48.0–44.0 (C_2-1_, C_2-3_, C_2-2_), 40.5 and 39.4 (CH_2_NHC(NH_2_)NH_2_^+^, C_1-3_, C_1-2_).

### 2.3. Preparation of GPEI-Functionalized oxCNTs

For the preparation of the nanohybrids oxCNTs@GPEI5K and oxCNTs@GPEI25K, 50 mg oxCNTs was dispersed in 50 mL dd H_2_O by ultrasonication for 30 min using a Hielscher UP200S high-intensity ultrasonic processor coupled with a sonotrode with a tip of 3 mm diameter (50% amplitude, 0.5 cycles/s). The pH was adjusted to ~9 by adding NaOH solution (0.5 M), and the dispersion was left under stirring for 24 h. Then, 150 mg GPEI5K or GPEI25K dissolved in 50 mL H_2_O was added to the above oxCNTs dispersion and stirred for further 48 h, at room temperature. The final products, oxCNTs@GPEI5K or oxCNTs@GPEI25K, were received after centrifugation at 25,000× *g*, washed until the pH of the supernatant reached the value of 6.5–7.0, and lyophilized.

### 2.4. Preparation of Rhodamine-Labeled GPEI-Functionalized oxCNTs

Rhodamine-labeled GPEI-functionalized oxCNT hybrids (oxCNTs@GPEI5K•Rh and oxCNTs@GPEI25K•Rh) were prepared by reacting the hybrids with rhodamine B isothiocyanate (Rh). Specifically, 15 mg of rhodamine B isothiocyanate, dissolved in methanol (2 mL), was added to 10 mg of oxCNTs@GPEIs dispersed in the same solvent (5 mL). The mixture was sonicated for 30 min and then was stirred in the dark, in inert atmosphere, at room temperature for 24 h. The final products, oxCNTs@GPEI5K•Rh and oxCNTs@GPEI25K•Rh, were received after centrifugation at 25,000× *g*, washing with methanol several times to remove the unreacted Rh, and subsequently vacuum-dried. The oxCNTs@GPEIs/rhodamine weight ratio in the final labeled hybrids was determined by UV–vis spectroscopy and found to be 5:1. In brief, the Rh content in a aqueous solution of a known oxCNTs@GPEIs•Rh concentration was calculated by recording the absorbance of the rhodamine moiety using a Cary 100 Conc UV–visible spectrophotometer (Varian Inc., Palo Alto, CA, USA) at 544 nm, employing a calibration curve constructed from standard Rh solutions (0.1–10 μg/mL).

### 2.5. Characterization of GPEI-Functionalized oxCNTs

^1^H and ^13^C NMR spectra were acquired at 25 °C on a Bruker Avance DRX spectrometer operating at 500 and 125.1 MHz, respectively, using D_2_O as solvent. The polymer content attached to the surface of oxCNTs was determined by ^1^H NMR using maleic acid as internal standard [64]. FTIR spectra in the region 500–4000 cm^−1^ were recorded on a Nicolet 6700 spectrometer (Thermo Scientific, Waltham, MA, USA) equipped with an attenuated total reflectance accessory with a diamond crystal (Smart Orbit, Thermo Electron Corporation, Madison, WI, USA) at 4 cm^−1^ resolution. One drop of oxCNTs@GPEIs solution in methanol was placed on the diamond element, and the solvent was removed under a steam of nitrogen to produce a thin film. Typically, for all FTIR spectra recorded, 128 scans were acquired and averaged. Raman spectra were acquired in the 400–2000 cm^−1^ range, employing a micro-Raman system RM 1000 Renishaw at an excitation wavelength at 532 nm (Nd-YAG). X-ray photoelectron spectroscopy (XPS) was applied using a SPECS GmbH spectrometer carrying a monochromatic MgKa source (hv = 1253.6 eV) and a Phoibos-100 hemispherical analyzer (Berlin, Germany). The spectroscopic experiments took place under ultrahigh vacuum at a base pressure of 4 × 10^−10^ mbar. The resolution was set to 1.16 eV to minimize measuring time. Spectral analysis included a Shirley background subtraction and a peak separation using mixed Gaussian–Lorentzian functions, in a least-squares curve-fitting program (WinSpec) developed at the Laboratoire Interdisciplinaire de Spectroscopie Electronique, University of Namur, Belgium. All binding energies were referenced to the C1s core level at 284.6 eV. Thermogravimetric analyses (TGA) were carried out employing a Setaram SETSYS Evolution 17 analyzer with a scanning rate of 5 °C/min under air atmosphere. Elemental analysis (EA) was implemented by a Perkin Elmer 240 CHN elemental analyzer. The morphology of GPEI-functionalized oxCNTs was studied by a Jeol JSM 7401F Field Emission Scanning Electron Microscope equipped with Gentle Beam mode (JEOL USA Inc., Peabody, MA, USA) and by a FEI Talos F200i field-emission (scanning) transmission electron microscope (Thermo Fisher Scientific Inc., Waltham, MA, USA) operating at 200 kV, equipped with a windowless energy-dispersive spectroscopy microanalyzer (6T/100 Bruker, Hamburg, Germany). For the preparation of TEM samples, a drop of oxCNTs@GPEIs aqueous solution (0.1 mg/mL) was dropped on a PELCO^®^ Formvar grid and was allowed to air dry.

### 2.6. Preparation and Characterization of GPEI-Functionalized oxCNT Aqueous Dispersions

For the preparation of the aqueous GPEI-functionalized oxCNTs suspensions, 1 mg of oxCNTs@GPEIs was dispersed into 1 mL distilled water by aid of ultrasonication for 30 min (Hielscher UP200S high-intensity ultrasonic processor equipped with a standard sonotrode having 3 mm tip diameter at 40% amplitude and 0.5 cycles/s). These stock dispersions were stable at room temperature for several weeks.

The aqueous dispersions of oxCNTs@GPEIs were characterized using UV–vis spectroscopy and *ζ*-potential. Specifically, UV–vis spectra of the oxCNTs@GPEIs aqueous dispersions (0.1 mg/mL) were recorded by a Cary 100 Conc UV–Visible spectrophotometer (Varian Inc., Mulgrave, Australia) in the range of 220–650 nm. *ζ*-potential measurements were carried out using a ZetaPlus (Brookhaven Instruments Corp, Long Island, NY, USA). In a typical measurement, 800 μL dispersions of oxCNTs@GPEIs (0.2 mg/mL) were diluted with water up to 1.6 mL, ten measurements were collected, and the results were averaged.

### 2.7. Preparation of DOX-Loaded GPEI-Functionalized oxCNTs

DOX loading was performed by dispersing 5 mg of oxCNTs@GPEIs in 5 mL aqueous DOX solution (1 mg/mL or 2 mg/mL, pH = 8.5), using ultrasonication for 30 min and further stirring at room temperature for 24 h. In order to remove non-bound DOX, the dispersions were centrifuged at 25,000 *g* for 45 min, and the precipitates were repeatedly rinsed, while the supernatants were collected for determination of DOX loading. The final DOX-loaded nanohybrids were obtained after lyophilization and were stored at 4 °C until use.

The amount of DOX loaded on oxCNTs@GPEIs was measured by a method analogous to the one described in the literature [65]. In brief, the DOX concentration of the supernatants, viz the concentration of the unloaded DOX, was determined using a Cary 100 Conc UV–visible spectrophotometer (Varian Inc.) at 480 nm calibrated against standard DOX solutions (2–30 μg/mL). Thus, the DOX loading on oxCNTs@GPEIs was calculated by the difference of the initial DOX concentration and the unloaded DOX concentration. The % DOX loading and efficiency on oxCNTs@GPEIs were calculated using the following Equations (1) and (2), respectively:

(1) $$\%\;DOX\;Loading=\frac{\mathrm{Weight}\;\mathrm{of}\;\mathrm{loaded}\;\mathrm{DOX}\;\left(\mathrm{mg}\right)\;}{\mathrm{Weight}\;\mathrm{of}\;\mathrm{oxCNTs}@\mathrm{GPEIs}\;\left(\mathrm{mg}\right)+\mathrm{Weight}\;\mathrm{of}\;\mathrm{loaded}\;\mathrm{DOX}\;\left(\mathrm{mg}\right)\;}*100$$

(2) $$\%\;DOX\;efficiency=\frac{\mathrm{Weight}\;\mathrm{of}\;\mathrm{loaded}\;\mathrm{DOX}\;\left(\mathrm{mg}\right)\;}{\mathrm{Weight}\;\mathrm{of}\;\mathrm{the}\;\mathrm{initial}\;\mathrm{DOX}\;\left(\mathrm{mg}\right)\;}*100$$

### 2.8. In Vitro pH-Dependent Release of DOX from GPEI-Functionalized oxCNTs

The release behavior of DOX from GPEI-functionalized oxCNTs was studied at two different pH conditions, i.e., 7.4 and 5.5. Thus, for these experiments 5 mg of GPEI-functionalized oxCNTs loaded with DOX was dispersed into 10 mL PBS (pH = 7.4) or acetate buffer (pH = 5.5) using sonication for 15 min and then incubated at 37 °C under mild agitation using a Stuart SI500 orbital shaker at approximately 200 rpm shaking speed. After each predetermined time interval, the dispersions were centrifuged at 15,000× *g*, 1 mL of the supernatant was withdrawn, and the released DOX concentration was measured using UV–vis as described above. For keeping the release medium volume constant, 1 mL of fresh buffer was added after each sampling, and the precipitate was re-dispersed using sonication for 5 min. The cumulative fraction of released DOX versus time was calculated using the Equation (3):

% DOX release = DOX_t_/DOX_initial_ × 100 (3)

where DOX_t_ is the DOX amount released at time t, and DOX_intial_ is the initial DOX amount loaded on GPEI-functionalized oxCNTs.

### 2.9. Cell Culture

Human prostate carcinoma DU145 and PC3 cell lines as well as normal human kidney HEK293 were obtained from the American Type Culture Collection (ATCC, Manassas, VA, USA). The cells were cultured in Dulbecco’s modified Eagle medium (D-MEM) with phenol red supplemented with 10% FBS, 1% penicillin/streptomycin and 1% L-glutamine at 37 °C in a 5% CO_2_ humidified atmosphere. All treatments were performed in complete medium.

### 2.10. Cell Viability Assay

The cytotoxicity of oxCNTs@GPEIs, DOX and DOX-loaded oxCNTs@GPEIs was assessed employing the MTT assay. DU145 or PC3 cancer cells and normal HEK293 cells were grown overnight at a density of 1 × 10^8^ cells/well into 96-well plates in complete medium. Cells were then treated for 3 h with free DOX, oxCNTs@GPEI (5 K or 25 K) nanocarriers and oxCNTs@GPEI (5 K or 25 K)-DOX, at various concentrations. After this period, the cell medium was removed and replaced with fresh medium, and then the cells were further cultured for 24 h and 48 h. MTT solution (1 mg/mL in D-MEM) was added to each well, cells were further incubated for 4 h, and the produced formazan crystals were solubilized with isopropanol. Their absorbance was measured at 540 nm in an InfiniteM200 microplate reader (Tecan group Ltd., Männedorf, Switzerland), and the relative cell viability was determined as cell survival percentage compared to untreated cells, which were used as control.

### 2.11. In Vitro Intracellular Uptake

The in vitro cellular uptake of DOX-loaded oxCNTs@GPEIs was assessed using flow cytometry and confocal laser scanning microscopy. For flow cytometry experiments, DU145 and HEK293 cells were grown overnight at a density of 50 × 10^4^ cells per well into 6-well plates in complete medium. Cells were then treated with free DOX and loaded DOX on oxCNTs@GPEI5K or oxCNTs@GPEI25K nanocarriers (oxCNTs@GPEI5K-DOX or oxCNTs@GPEI25K-DOX) as well as with unloaded nanocarriers for 1, 3 and 5 h. In all experiments, the DOX concentration was 1 μM, and the corresponding nanocarriers’ concentration was 2 μg/mL. After the treatment, the cells were rinsed twice with 1× PBS, trypsinized, and the resulting cell suspensions were centrifuged for 5 min at 1000 rpm. Finally, the collected cells were re-suspended in 1× PBS, and the DOX uptake was analyzed using a FACSCalibur flow cytometer (Becton Dickinson, Heidelberg, Germany) [66].

For confocal experiments, DU145 and HEK293 cells were grown overnight at a density of 5 × 10^5^ cells on poly-D-lysine coverslips in complete medium. The cells were subsequently incubated with free DOX (3 μΜ), oxCNTs@GPEI (5 K or 25 K) labeled with rhodamine (2 μg/mL) and oxCNTs@GPEI (5 K or 25 K)-DOX (1 μΜ DOX concentration) for 3 h. Then, cells on coverslips were rinsed with 1× PBS, fixed with 4% paraformaldehyde/1× PBS for 20 min at room temperature and rinsed again with 1× PBS. For nuclei visualization, cells were incubated with TO-PRO-3 for 5 min. Finally, cells on coverslips were mounted with mounting medium (Dako, Denmark) and were observed under a Leica TCS SP8 MP multiphoton confocal microscope (Wetzlar, Germany). Images were acquired with the spectral detector of the microscope using appropriate emission wavelength ranges; DOX and rhodamine (red color) were excited at 561 nm, and emission was recorded between 570 and 650 nm, while TO-PRO-3 was excited at 642 nm, and emission was recorded at 661 nm (far red, pseudo-color blue). Images were acquired with LAS X software (Leica Microsystems CMS GmbH, Wetzlar, Germany) and were processed with Image J software.

### 2.12. Necrosis/Apoptosis Analysis Using Flow Cytometry

The necrosis/apoptosis ratio of DU145 cells upon treatments was determined using Annexin V-FITC/7-Aminoactinomycin D (7-AAD) double staining. Briefly, DU145 cells grown in 6-well plates overnight were treated with free DOX, oxCNTs@GPEI (5 K or 25 K) and oxCNTs@GPEIs-DOX for 3 h. In all experiments, the DOX concentration was 1 μM, and the corresponding nanocarriers’ concentration was 2 μg/mL. Following treatments, the cells were trypsinized, suspended in 1× PBS buffer and centrifuged for 5 min at 1000 rpm. Finally, the cell pellet was re-suspended in 100 μL buffer and 5 μL of Annexin V-FITC, and 5 μL 7-AAD solution was added. Following 15 min incubation in the dark, the cells were analyzed with a FACSCalibur flow cytometer (Becton Dickinson, Heidelberg, Germany).

### 2.13. Statistical Analysis

At least three independent repetitions for each experiment were performed, and the data are presented as mean ± standard deviation. A student’s *t*-test was employed to assess statistical significance for all treatments (* *p* < 0.05, ** *p* < 0.01, *** *p* < 0.001).

## 3. Results and Discussion

### 3.1. Synthesis and Characterization of GPEI-Functionalized oxCNTs

**Scheme 1:**
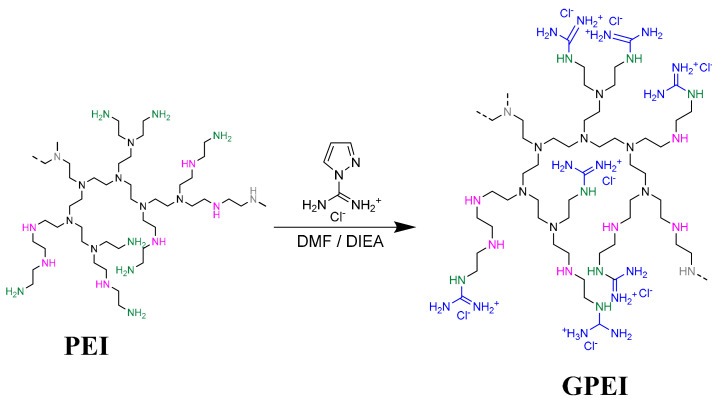
Schematic representation of polyethyleneimine (PEI) and the reaction scheme of the guanidinylation.

Hyperbranched polyethyleneimine (PEI) was fully functionalized at the primary amino groups with guanidinium groups following a method analogous to that described in the literature [62,63]. Specifically, the primary amino groups of PEI with molecular weights of 5000 and 25,000 Da were reacted with an appropriate amount of 1*H*-Pyrazole-1-carboxamidine hydrochloride in an alkaline environment, affording two guanidinylated PEI derivatives, GPEI5K and GPEI25K (Scheme 1). Their chemical structures were characterized by ^1^H and ^13^C NMR spectroscopy. More detailed, the successful introduction of guanidinium groups to PEI was established by the presence in the ^1^H NMR spectra of both guanidinylated derivatives (Supplementary Material Figure S1) of a new peak at 3.25 ppm attributed to the protons of α-CH_2_ groups relative to guanidinium groups, while a multiplet in the region between 2.50 and 2.70 ppm was attributed to the protons of methyl groups of PEI scaffold. Comparing the integration of peaks at 3.25 ppm and 2.70–2.50 ppm, the successful introduction of guanidinium groups to the primary amino group of PEI was confirmed at approximately 100% degree of substitution (98% for GPEI5K and 95% for GPEI25K). Furthermore, the successful attachment of guanidinium groups to the primary amino groups of PEI was confirmed by the presence in the ^13^C NMR spectra of both guanidinylated derivatives (Figure S2), of new peaks at 157.0 ppm, attributed to the guanidinium group carbon, and at 39.4 and 40.5 ppm assigned to the α-methylene groups (C_1-3_ and C_1-2_) relative to the guanidinium group, respectively.

**Scheme 2:**
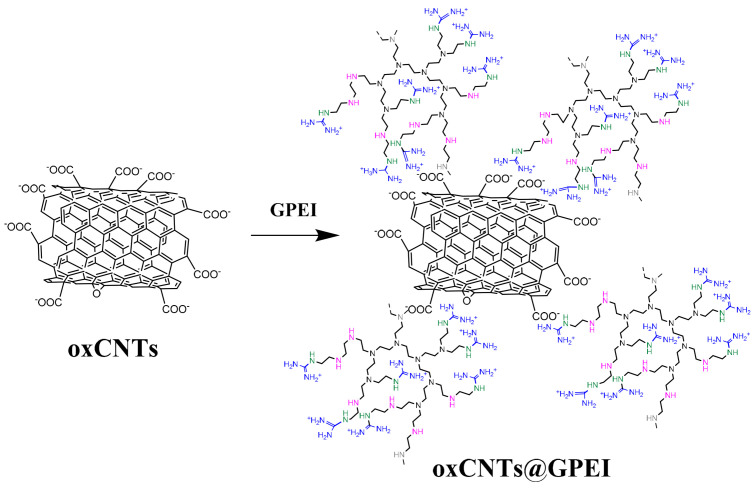
Schematic representation of GPEI-functionalized oxCNTs.

Using the as-prepared polyethyleneimine derivatives, oxidized multi-walled nanotubes were decorated with positive-charged guanidinium moieties by a simple non-covalent modification procedure (Scheme 2). It is known that guanidinium groups strongly interact with carboxylate, phosphate or sulfate groups both through electrostatic interactions and bidentate hydrogen bonds due to their electronic and geometrical complementarity [35,36,67]. Additionally, as mentioned in previous works, PEI weakly interacts with CNTs and mainly with the oxidized CNTs through electrostatic and CH-π interactions, leading to the efficient PEI wrapping around the CNTs’ sidewalls [68,69,70,71]. Thus, in this work, a combination of the bidentate hydrogen bonds and the electrostatic interaction between the guanidinium groups of GPEIs and the carboxylate groups of oxCNTs together with the physisorption of the PEI scaffold on CNTs’ sidewalls takes place, leading to the formation of two hybrid materials, oxCNTs@GPEI5K and oxCNTs@GPEI25K, which were subsequently characterized by a variety of techniques, such as XPS, FTIR, RAMAN, SEM, TEM, etc.

The successful introduction of GPEIs on the oxCNTs’ surface was initially established by X-ray photoelectron spectroscopy. Using this technique, information about the type of interactions taking place after the functionalization of oxCNTs with GPEI was obtained, revealing the successful synthesis of oxCNTs@GPEIs. The XPS survey spectra of oxCNTs@GPEIs (Figure S3) exhibited not only the characteristic O1s and C1s contributions mainly from the oxCNTs due to its graphitic framework, but also two additional peaks centered at binding energies of ~400 eV and ~200 eV, characteristic of nitrogen and chloride anions, respectively. These peaks exclusively originate from GPEI derivatives since they are absent in the reference survey spectrum of oxCNTs, while present in the survey spectrum of GPEIs (unpublished material) [72]. Therefore, the presence of the nitrogen-rich GPEIs in the final hybrids is clearly observed. Moreover, quantitative information can be obtained by analyzing these peaks. Specifically, the atomic percentages of the elements taking part in the interaction show that the oxCNT@GPEI25K possesses a higher nitrogen amount than that of oxCNT@GPEI5K i.e., 8.4% for oxCNT@GPEI25K instead of 4.0% for oxCNT@GPEI5K (Table S1).

Further information regarding the chemical environment of oxCNTs@GPEIs can be obtained from the C1s and N1s core level XPS spectra. The high-resolution C1s and N1s photoelectron spectra of guanidinylated hyperbranched polyethyleneimine derivatives as well as the high-resolution C1s of oxCNTs were included as revealed in Figures S4 and S5. More specifically, the C1s spectrum for both GPEI5K (Figure S4a) and GPEI25K (Figure S4b) deconvoluted into four fitted peaks representing the C-C bonds of the PEI scaffold as well the C-N and C=N bonds due to the ethyleneimine (C-N) and guanidinium groups (C=N), revealing the successful introduction of guanidinium groups to PEI. Additionally, another fitted peak was observed centered at 288.9 eV in the C1s spectra of GPEIs, attributed to the protonated guanidinium groups (C=N^+^) [73]. From the N1s peak, the fitted curves revealed the different functional groups of the final products (Figure S4c,d). At 398.9 eV we observed the =N- bonds revealing the successful incorporation of guanidinium groups, while at 399.9 we had the –NH- bonds of PEI. Finally, at higher binding energies (401.0 eV), we received a contribution as a result of protonated amines of guanidinium groups or/and PEI. Moreover, the high-resolution C1s spectrum of oxCNTs can be fitted using four components as shown in Figure S5. The component at 284.7 eV was attributed to the C-C bonds due to the graphitic structure representing the 54.9% of the whole carbon amount, while a second peak at 285.7 eV was due to C-O bonds (23.4%). Finally, the contributions at 287 eV and 288.6 eV were assigned to oxCNTs epoxy and carboxyl groups, respectively, revealing the successful implementation of the oxidation process.

**Figure 1:**
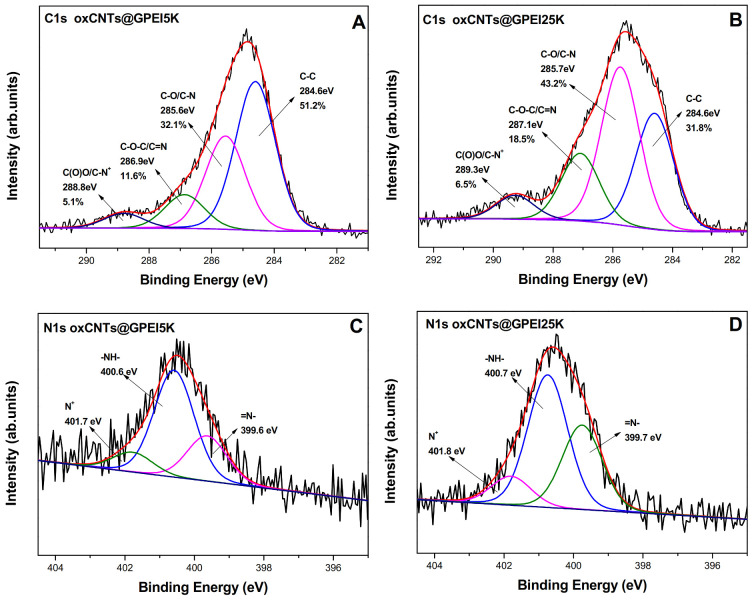
XPS high-resolution spectra for the C1s (**A**,**B**) and N1s (**C**,**D**) of oxCNT@GPEI5K and oxCNT@GPEI25K.

Furthermore, the corresponding C1s photoelectron spectra of oxCNT@GPEIs was also fitted using four components as presented in Figure 1A,B. Comparing these spectra with those of oxCNTs, an important increase in the overall carbon intensity from 23.4% to 32.1% for oxCNT@GPEI5K was observed in the intensity of the peak at 285.7 eV, representing the C-O/C-N bonds, reaching 43.2% for oxCNT@GPEI25K. A similar behavior was observed for the intensity of the 288.8 eV peak, responsible for C(O)O/C-N^+^ bonds, which increased from 4.2% to 5.1% of the overall carbon intensity for oxCNT@GPEI5K, reaching 6.5% for oxCNT@GPEI25K. These changes are attributed to the introduction of C-N and C-N^+^ bonds, both originating from GPEIs. It should be noted that these peaks were more intense for oxCNT@GPEI25K than oxCNT@GPEI5K (43.2% instead of 32.1%, and 15.1% instead of 10.6%, respectively), due to the higher GPEI25K content in the oxCNT@GPEI25K hybrid. Moreover, a decrease in intensity of the peak at 286.9 eV attributed to C-O-C/C=N bonds was observed due to interaction of oxCNTs epoxy groups with guanidinium moieties. Additionally, from the N1s XPS fitted peaks of both oxCNT@GPEI5K and oxCNT@GPEI25K (Figure 1C,D), a total shift of about 0.8–1.0 eV was detected for the three fitted peaks compared to the N1s spectra of GPEIs (Figure S4c,d). A peak shift as large as 1 eV was explained by the synergistic effect of carboxyl ions and the donating electrons or/and by hydrogen bonding interactions between the oxidized CNTs oxygenated groups and the nitrogen groups of GPEI.

**Figure 2:**
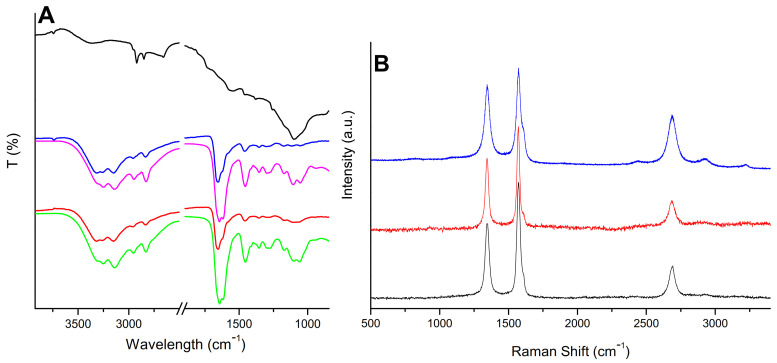
FTIR (**A**) and Raman (**B**) spectra of oxCNTs (black), GPEI5K (green), GPEI25K (magenta), oxCNTs@GPEI5K (red) and oxCNTs@GPEI25K (blue).

The findings from the XPS analysis are further confirmed by FTIR studies. Figure 2A shows the GPEIs, oxCNTs and GPEI-functionalized oxCNTs FTIR spectra. For oxCNTs, a C=C stretching band at 1650 cm^−1^ can be attributed to the CNTs graphite structure, while the presence of the oxygen-containing groups (carboxylates, carbonyl, hydroxyl and epoxy groups) on the CNTs’ surface can be confirmed by the existence of a C=O stretching band at 1740 cm^−1^, a broad OH stretching band centered at 3370 cm^−1^, a strong C-OH stretching band at 1100 cm^−1^, a band at 1255 cm^−1^ attributed to stretching vibrations of C–O–C as well as two peaks associated with the carboxylate anion stretch mode at 1565 and 1380 cm^−1^ (asymmetrical and symmetrical vibrations of COO-, respectively) [74]. Additionally, the FTIR spectra of both GPEIs exhibited the sample characteristic bands: the stretching vibration of primary and secondary amino groups of guanidinium moieties at 3260 and 3140 cm^−1^; the asymmetrical and symmetrical stretching vibrations of CH_2_ at 2950 and 2830 cm^−1^, respectively; the symmetrical and asymmetrical stretching vibrations of guanidinium groups (C=N) at 1644 and 1619 cm^−1^, respectively; the bending mode of CH_2_ at 1455 cm^−1^ and the asymmetrical and symmetrical vibrations of C-N at 1105 and 1050 cm^−1^, respectively [75]. Comparing the FTIR spectra of oxCNTs and GPEIs to that of GPEI-functionalized oxCNTs (Figure 2A), the presence of both oxCNTs and GPEIs in the hybrid materials was obvious, suggesting their successful interaction.

The successful functionalization of oxCNTs was also confirmed using Raman spectroscopy. The oxCNTs and GPEIs-functionalized CNTs spectra are presented in Figure 2B. Both spectra exhibited two intense graphite bands at 1345 cm^–1^ and 1585 cm^–1^. The band at 1585 cm^–1^ was attributed to the tangential vibrations of the C–C bonds in the well-ordered graphite layers (G-band), while the band at 1345 cm^–1^ was assigned to the presence of disorders in the sp^2^ carbon network (D-band). Additionally, G’″-band, attributed to the D-band overtone, was observed at ~2700 cm^–1^. It is known that the intensity of the D-band is strongly related to the structural disorder due to amorphous carbon and defects present on CNTs sidewalls. The D- and G- bands relative intensity ratio (I_D_/I_G_) is widely used as a measure of the defects occurring during functionalization of the nanotube walls [76]. The GPEIs functionalization led to a slight increase in the I_D_/I_G_ ratio from 0.73 to 0.81 for oxCNTs@GPEI5K and 0.92 for oxCNTs@GPEI25K, suggesting that GPEI derivatives successfully wrapped all over the sidewalls of CNTs without causing any significant damage to the CNTs graphite structure. Analogous results were reported when multi-walled carbon nanotubes were functionalized with PEI [77] or quaternized PEI derivatives [78] through non-covalent bonds, indicating that after successful interaction, the CNTs surface remained almost intact.

**Figure 3:**
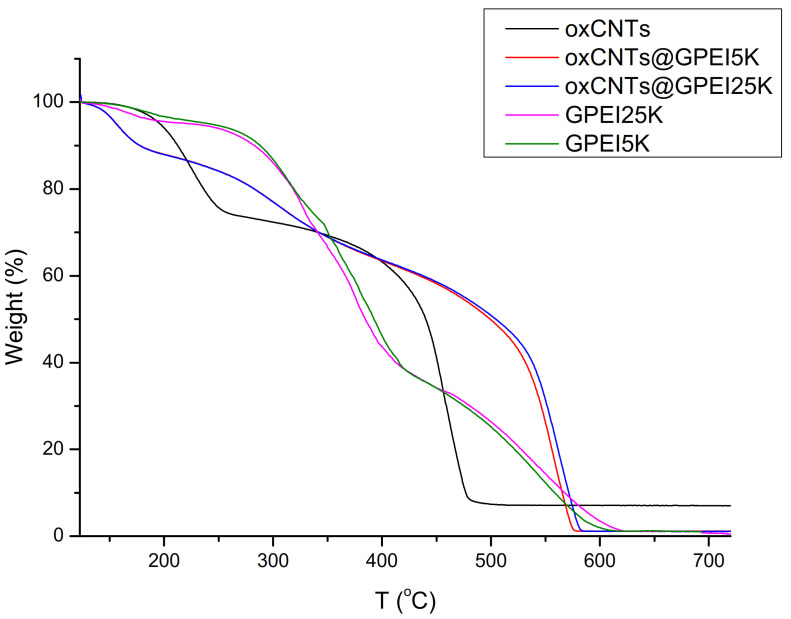
Thermogravimetric analysis (TGA) curves of oxCNTs and GPEI-functionalized oxCNTs.

Information regarding the presence of GPEI on the surface of oxCNTs was obtained via thermogravimetric analysis (TGA). The weight loss curves of oxCNTs and GPEI-functionalized oxCNTs are shown in Figure 3. For oxCNTs, two distinct decomposition regions were observed, one in the temperature range of 100–240 °C, recording a weight loss of ~20% of the initial weight due to the removal of oxygen-containing functional groups present on the oxCNTs, and another one at higher temperatures (>350 °C), ascribed to the thermal degradation of the graphitic lattice. On the other hand, in the TGA curves of GPEI-functionalized oxCNTs, a slight weight loss (~10%) up to 300 °C as well as a higher one (~30%) up to 400 °C were detected, due to both the removal of oxCNTs’ oxygen-containing groups and the partial GPEI degradation. The weight loss for GPEI-functionalized oxCNTs at temperatures higher than 400 °C was attributed not only to the decomposition of graphitic lattice, but also to the GPEI thermal destruction. Moreover, above ~550 °C, a sharp weight loss was observed, indicating the total thermal degradation of the graphitic framework. It is obvious that the presence of GPEIs on the oxCNTs surface delayed the thermal decomposition of CNTs, providing further evidence of the successful functionalization of oxCNTs with GPEIs. Unfortunately, TGA results cannot be used for the quantification of the polymer content, due to the simultaneous decomposition of both oxCNTs and GPEIs, and can only provide qualitative evidence that functionalization had taken place. Hence, in order to determine the GPEIs content as well as to confirm GPEIs’ presence in oxCNTs@GPEI5K and oxCNTs@GPEI25K hybrids, ^1^H NMR spectroscopy was applied using maleic acid as an internal standard [64]. From the ^1^H NMR spectra of oxCNTs@GPEI5K and oxCNTs@GPEI25K (Figure S6), the presence of GPEIs in the hybrids was confirmed (peaks at 3.25 ppm and 2.70–2.50 ppm attributed to the protons of GPEI as mentioned before). Additionally, by comparing the integrals of the peaks at (i) 6.35 ppm attributed to the protons of methine groups of maleic acid and (ii) 2.70–2.50 ppm attributed to the protons of methyl groups of PEI scaffold, one can calculate that, on average, 32 μmol (0.214 g) GPEI5K and 8.5 μmol (0.284 g) GPEI25K were attached to 1 g of oxCNTs@GPEI5K and oxCNTs@GPEI25K, respectively. Elemental analysis further supported the results of ^1^H NMR spectroscopy. As previously mentioned in the literature [78,79], the nitrogen signal in the functionalized oxCNTs mainly originates from GPEI. Therefore, the quantity of GPEIs attached to the oxCNTs can be calculated by comparing the nitrogen signal of the final materials to that of the starting oxCNTs using the Equation (4):

GPEI (% *w/w*) = (N_s_ − N_oxCNTs_)/(N_GPEI_ − N_oxCNTs_) * 100 (4)

where N_s_, N_GPEI_ and N_oxCNTs_ are the nitrogen elemental mass fraction in GPEI-functionalized oxCNTs, GPEI and oxCNTs, respectively. The obtained results are summarized in Table S1, where the actual values of the GPEI weight fraction in oxCNTs@GPEI5K and oxCNTs@GPEI25K were calculated at 22.7% and 27.5%, respectively, in line with the findings from ^1^H NMR.

**Figure 4:**
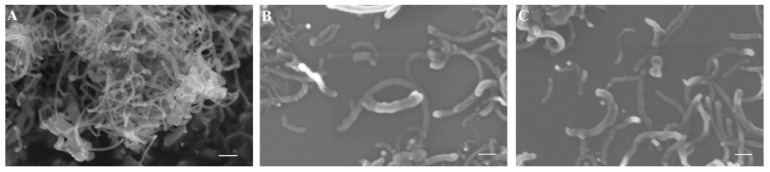
SEM images of oxCNTs (**A**), oxCNTs@GPEI5K (**B**) and oxCNTs@GPEI25K (**C**). The scale bar is 200 nm.

The morphology of oxCNTs after their functionalization with GPEIs was studied by scanning electron (SEM) and high-resolution transmission electron (HRTEM) microscopies. As shown in Figure 4A, oxCNTs appeared to be tangled, while some of them formed agglomerates. On the other hand, although the morphology of both GPEI-functionalized oxCNTs did not change significantly compared to the oxCNTs, they were shown to be de-bundled with no aggregation of nanotubes observed (Figure 4B,C). Additionally, their surface was not as smooth as in the case of oxCNTs, revealing successful polymer wrapping all over the sidewalls of oxCNTs.

**Figure 5:**
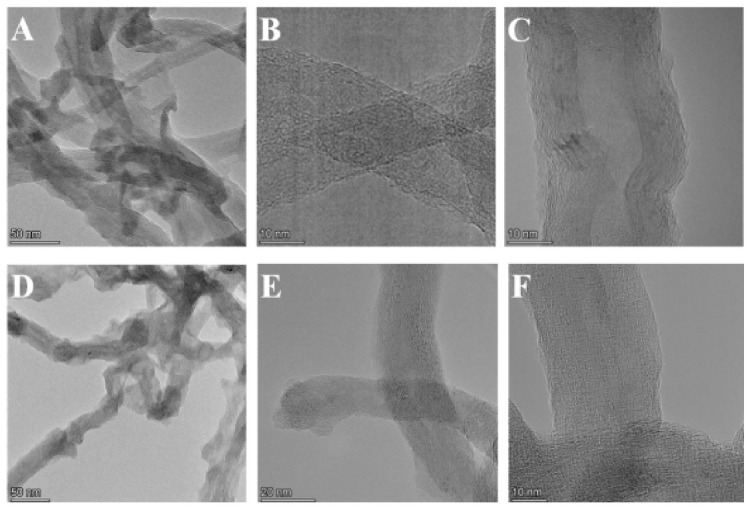

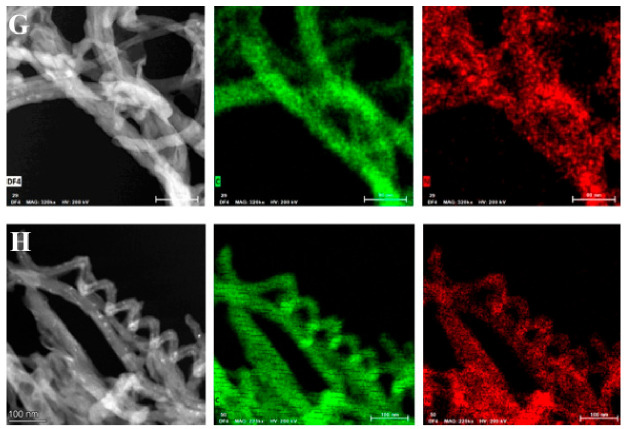
HRTEM images of oxCNTs@GPEI5K (**A,B,C**) and oxCNTs@GPEI25K (**D,E,F**). Bright-field TEM images showing the morphology of oxCNTs@GPEI5K (**G**) and oxCNTs@GPEI25K (**H**), followed by dark-field (DF4 detector) STEM images and the corresponding EDS elemental mapping images of C (K edge) and N (K edge).

Moreover, the morphology of GPEI-functionalized oxCNTs and the presence of GPEIs on sidewalls of oxCNTs were investigated by a combination of high-resolution TEM (HRTEM) and dark-field (DF4 detector) STEM imaging with the corresponding energy-dispersive X-ray (EDX) mapping images. As shown in Figure 5, the structured graphite walls of oxCNTs can be observed, fully covered by an amorphous layer of polymer. Further evidence of the presence of GPEIs on the sidewalls of oxCNTs can be obtained by the determination of the spatial distribution of carbon and nitrogen from the EDS mapping images of C (K edge) and N (K edge). From the element mapping images of oxCNTs@GPEI5K (Figure 5G) and oxCNTs@GPEI25K (Figure 5H), it is obvious that the nitrogen that exclusively originates from GPEI was uniformly anchored on the same position where carbon was also situated, revealing that oxCNTs were uniformly covered by GPEI.

### 3.2. Evaluation of Aqueous Dispersion of GPEI-Functionalized oxCNTs

**Figure 6:**
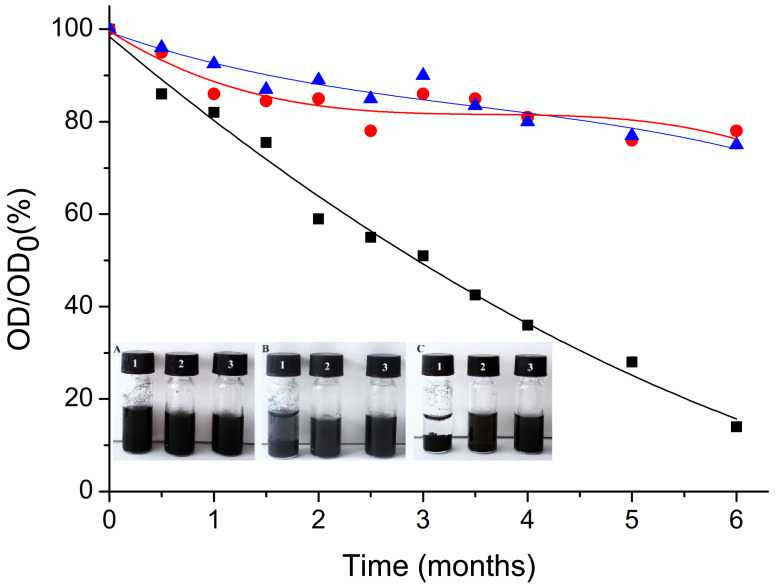
Sedimentation behavior of aqueous dispersions (1 mg/mL) of oxCNTs (black), oxCNTs@GPEI5K (red) and oxCNTs@GPEI25K (blue) at different aging times. Insertion: Dispersion state of oxCNTs (1), oxCNTs@GPEI5K (2) and oxCNTs@GPEI25K (3) in water (1 mg/mL), immediately after sonication (**A**) and after quiescent settling for 1 month (**B**) and 6 months (**C**).

The successful modification of oxCNTs with GPEIs was also confirmed by the increase in the aqueous dispersibility of oxCNTs after functionalization. Aqueous dispersions containing oxCNTs and GPEI-functionalized oxCNTs at a concentration of 1.0 mg/mL were prepared and observed for 6 months. The dispersion states of these CNTs samples in aqueous media are shown in Figure 6 (insertion) immediately after sonication (A) and after quiescent settling for 1 month (B) and 6 months (C). It is obvious that the aqueous stability of oxCNTs was poor since oxCNTs had started to flocculate within 1 month after the sonication process, while after settling for 6 months all the oxCNTs had precipitated. This behavior was attributed to the strong tendency of CNTs to aggregate in aqueous media due to their high surface energy that drives them to agglomerate into large bundles [80]. Many attempts have been made to increase the aqueous dispersibility of CNTs such as modification of their surface using various oxidation processes [21], or dispersing them into aqueous solutions containing various surfactants [25,26] or polymers [22,23,24] at high concentrations, but the resulting CNTs dispersions were only stable for a short time. Herein, positively charged GPEIs were used to functionalize the negatively charged oxidized CNTs through electrostatic interactions and van der Waals attraction forces, yielding oxCNTs decorated with high positive charge moieties, which can successfully form stable aqueous dispersions. As revealed by visual observation over time (Figure 6, insertion), both GPEI derivatives improved the dispersibility of oxCNTs in water since the dispersions of GPEI-functionalized oxCNTs obtained, even after quiescent settling for six months, remained stable, without the presence of any flocculation. These enhanced dispersion properties can be attributed to the presence of guanidinium groups on the surface of the oxCNTs, which can induce the hydrophilicity of oxCNTs, providing high aqueous compatibility, while avoiding the agglomeration of CNTs due to electrostatic repulsion. Similar behavior was also observed when oxCNTs were decorated using quaternized PEI derivatives [78]. The obtained hybrid derivatives can efficiently disperse in aqueous media due to the presence of the hydrophilic quaternary ammonium groups on oxCNTs’ surface, giving the CNTs’ aqueous compatibility. On the contrary, non-covalent functionalization of oxCNTs with the parent PEI derivatives, PEI5K and PEI 25K, afforded hybrid materials (oxCNTs@PEI5K and oxCNTs@PEI25K) with low aqueous stability (flocculation was observed within half an hour after the sonication process) probably due to the PEI hyperbranched structure that induces modification of the CNTs’ electronic structure due to the reduction in electrostatic interactions. Analogous results were obtained when single-walled carbon nanotubes were functionalized with PEI derivatives [68].

Furthermore, the colloidal stability of oxCNTs was evaluated employing UV–vis spectroscopy, using the characteristic absorption of CNTs at 263 nm [81]. As already known from the literature, as opposed to individual CNTs, bundled CNTs are not active in the UV–vis region [82]; hence, UV–vis spectroscopy can be used to investigate their dispersibility. The reduction in optical density (O.D.) of oxCNTs and GPEI-functionalized oxCNT aqueous dispersions within the storage periods can be seen in Figure 6. Both GPEI-functionalized oxCNTs exhibited noticeable stability for at least six months, with a mere ~20% reduction in optical densities compared to the initial O.D. In contrast, for oxCNTs, O.D. reduced by 90% after quiescent settling for six months. This behavior can be attributed to the gradual flocculation of CNTs that finally precipitate. These results are in agreement with the visual inspection of the CNT dispersions shown in Figure 6 (insertion). To the best of our knowledge the aqueous stability attained after functionalization with GPEIs, a crucial parameter for a drug delivery system, is one of the highest reported in the literature.

Moreover, the successful attachment of GPEIs on the surface of oxCNTs was validated by zeta potential measurements. As expected, the *ζ*-potential value of the oxCNTs dispersion at pH = 7.0 was estimated around −30.6_±1.4_ mV, due to the negative oxygen-containing groups on the oxCNTs surface. On the contrary, the *ζ*-potential values of the GPEI-functionalized oxCNTs dispersions were found to be significantly more positive, i.e., approximately +50.4_±1.0_ mV and +54.9_±0.8_ mV for oxCNTs@GPEI5K and oxCNTs@GPEI25K, respectively, revealing the successful decoration of the oxCNTs surface with the positively charged guanidinium groups deriving from GPEIs molecules. It should be noted that all *ζ*-potential values were greater than +30 mV, indicating stable aqueous colloidal suspensions, where electrostatic repulsion caused by very high surface charges resulted in strongly repulsive double-layer force that inhibited CNTs’ aggregation, in agreement with the previously mentioned results [83]. This is also in line with the findings related to the poor aqueous stability of the functionalized oxCNTs with the parent PEI derivatives, whose *ζ*-potential values were found to be just over +30 mV (+31.2_±1.1_ mV for oxCNTs@PEI5K and +33.5_±1.2_ mV for oxCNTs@PEI25K), much lower than the corresponding oxCNTs@GPEI5K and oxCNTs@GPEI25K values.

### 3.3. In Vitro DOX Loading and pH-Dependent Release

DOX loading was performed under basic conditions by simple mixing of oxCNTs@GPEIs dispersions with aqueous DOX solution at weight ratios 1:1 and 1:2. The DOX loading of oxCNTs@GPEIs and DOX efficiency were spectroscopically determined using UV–vis and calculated according to Equations (1) and (2). The oxCNTs@GPEI5K and oxCNTs@GPEI25K DOX loading contents were 43.0% and 62.5%, respectively, and the corresponding DOX efficiencies were 77.1% and 98.6% when the initial DOX concentration was 1 mg/mL. Upon increase in initial DOX concentration to 2 mg/mL, the DOX efficiency increased to 78.7% for oxCNTs@GPEI5K and 99.5% for oxCNTs@GPEI25K, while the corresponding DOX loadings were 44.5% and 51.7%. It should be noted that under similar experimental conditions, the DOX loading content of oxCNTs was much lower (35% and 47%, when the initial DOX concentration is 1 and 2 mg/mL, respectively) compared to the corresponding contents of oxCNTs@GPEIs. This is probably attributed to the presence of GPEIs on the sidewalls of oxCNTs, since it is known that dendritic polymers and their derivatives can efficiently encapsulate various active ingredients such as doxorubicin [31,84].

**Figure 7:**
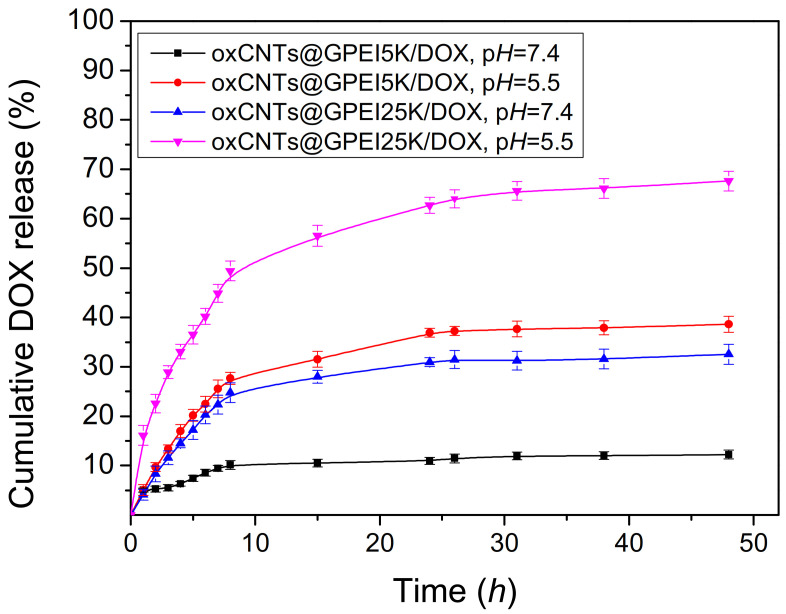
Cumulative release profiles of DOX from oxCNTs@GPEI5K and oxCNTs@GPEI25K in PBS (pH = 7.4) and acetate buffer (pH = 5.5).

The in vitro DOX release from both oxCNTs@GPEI5K and oxCNTs@GPEI25K systems was investigated in two different pH conditions, i.e., pH = 7.4 and 5.5, in order to simulate the physiological condition and the lysosomal pH or the acidic extra-tumor environment [85], respectively. The DOX release profiles from both systems at pH = 7.4 and 5.5 are shown in Figure 7. As observed, only 10% DOX from oxCNTs@GPEI5K and 27% from oxCNTs@GPEI25K was released at an almost linear rate in the first 8 h, under physiological pH (pH = 7.4), followed by a relatively slow release of about 2% and 10%, respectively, in the next 40 h. On the contrary, DOX was released from both systems at a significantly faster rate under acidic pH (pH = 5.5), since 27% (oxCNTs@GPEI5K) and 50% (oxCNTs@GPEI25K ) DOX was released with a steady rate in the first 8 h with a corresponding release of 12% and 17% DOX, respectively, in the next 40 h. Moreover, it is obvious that DOX can be released from oxCNTs@GPEI25K at a significantly faster rate at both tested environments compared to oxCNTs@GPEI5K. Being a hydrophobic weak base (p*K_a_* = 8.2–8.3) [86], DOX is partially deprotonated at physiological pH, favoring π–π stacking interactions with MWCNTs [50,58]. Therefore, at this pH it remains well bound on the nanocarriers. In contrast, under a mildly acidic environment (pH = 5.5) the protonated form of DOX prevails, prompting its fast release. Analogous results were reported by other research groups [58,59], for similar pH conditions simulating the extra-tumor environment or the micro-environment of intracellular lysosomes or endosomes. DOX was released from functionalized multi-walled carbon nanotubes at a significantly slower rate under physiological pH compared to mildly acidic conditions. This pH-dependent release behavior is expected to be beneficiary for antitumor treatment, since DOX is expected to be ineffective against normal tissues, while it may be released from the nanocarriers at the targeted tumor sites.

### 3.4. In Vitro Anticancer Activity of DOX-Loaded GPEI-Functionalized oxCNTs

**Figure 8:**
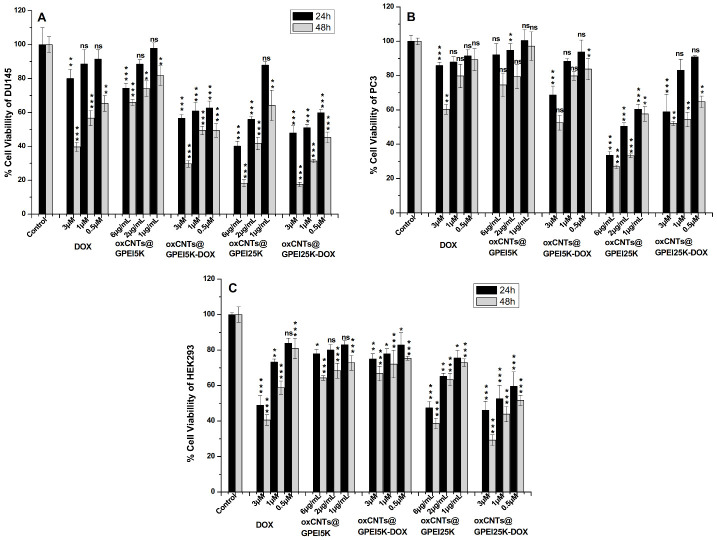
Comparative toxicities of oxCNTs@GPEI5K and oxCNTs@GPEI25K nanocarriers, oxCNTs@GPEI5K- DOX, oxCNTs@GPEI25K–DOX, and free DOX on carcinoma DU145 (**A**) and PC3 (**B**) cell lines as well as in normal HEK293 cells (**C**) following incubation at various concentrations for 3 h as determined by MTT assays 24 and 48 h following incubation. In all experiments, the DOX concentrations were 0.5, 1 and 3 μM, and the corresponding nanocarriers’ concentrations were 1, 2 and 6 μg/mL. Significance was calculated with the Student t-test. * *p* > 0.05, ** *p* > 0.01, *** *p* > 0.001, while no annotation implies no statistical significance (ns), *p* > 0.05.

Effective drug delivery is an important strategy to enhance the therapeutic effectiveness of cancer treatment while avoiding the side effects caused by anticancer drugs. Cancerous PC3 and DU145 cells and non-cancerous HEK293 cells were used to evaluate the specific therapeutic efficacy of DOX-loaded GPEI-functionalized oxCNTs. Thus, the cytotoxicity of DOX-loaded GPEI-functionalized oxCNTs compared to free DOX was assessed by the MTT method. As shown in Figure 8, 24 h treatment with free DOX in cancer cell lines PC3 and DU145 did not cause significant cytotoxicity (80–90% cell viability) at neither low nor high concentrations compared to the control, whereas 48 h treatment resulted in higher cytotoxicity in DU145 (~40% cell viability at the highest tested DOX concentration of 3 μM), compared to more resistant PC3 cells (~60% cell viability at the highest tested DOX concentration of 3 μM). The latter is consistent with other studies, where DU145 were found to be more sensitive to DOX compared to PC3 cells [87]. On the contrary, normal HEK293 were shown to be greatly affected by both 24 h and 48 h DOX treatment (~40–50% cell viability at the highest tested DOX concentration of 3 μM), which is also in accordance with the literature, where treatment with free DOX results in greater cytotoxicity in normal cells, compared to resistant cancer cell lines [88]. Instead, when DOX is loaded on the oxCNTs@GPEI5K carrier, the DU145 cell viability is greatly reduced, showing a progressive toxicity versus concentration with a maximum cytocidal activity of c.a. ~70% after 48 h incubation (~30% survival). In contrast, the administration of oxCNTs@GPEI5K-DOX on more resistant PC3 cells showed that cell viability was affected only at high concentrations. Notably, after 24 and 48 h of treatment with the oxCNTs@GPEI5K-DOX system, HEK293 cell viability remained mostly unaffected. This demonstrates that the efficiency of loaded DOX significantly increased with specificity to cancer cells. Importantly, the oxCNTs@GPEI5K nanocarrier exhibited almost no cytotoxic effects on either normal or cancer cell lines at the tested concentrations (~75% cell viability at the highest tested concentration of 6μg/mL after 48 h incubation), suggesting that this system could be used as a safe drug delivery platform.

Conversely, treatment with the oxCNTs@GPEI25K-DOX system resulted in high cytotoxicity against all cell lines and more prominently against DU145 and HEK293 cells after a 48 h incubation time (Figure 8). However, contrary to oxCNTs@GPEI5K, 24 h and 48 h treatment with oxCNTs@GPEI25K greatly reduced cell viability in all cell lines in a concentration-dependent manner, regardless of DOX, indicating that it can be used for biological applications only at low concentrations (~60% survival of DU145 and PC3, ~80% survival of HEK293 at a concentration of 1 μg/mL after 48 h incubation). Moreover, to confirm that oxCNTs@GPEI25K system’s cytotoxic properties are not solely due to their oxCNTs or PEI content but to their combination, the toxicity of oxCNTs and GPEI was individually assessed against DU145 cells in concentrations analogous to those contained in oxCNTs@GPEIs, head-to-head with parent PEIs in equivalent concentrations. As observed in Figure S7, oxCNTs demonstrated a mild toxicity after 24 h treatment (~70% cell viability at the highest tested concentration). Analogous results, reported in the literature, revealed that oxCNTs exhibited cytotoxicity against various cell lines due to the membrane damage and to the generation of reactive oxygen species and oxidative stress inducing cell apoptosis [89,90,91]. Interestingly, both GPEI5K and GPEI25K showed significant cytocidal activity (50% and 60% survival at concentration almost equal to that contained in 6 μg/mL of oxCNTs@GPEI5K and oxCNTs@GPEI25K, respectively, vis. 1.5 μg/mL), with GPEI25K being significantly more cytotoxic (Figure S7). Comparing the toxicities of both GPEI derivatives with those of the parent PEIs, it is obvious that both GPEIs were more toxic. This can be attributed to the more efficient internalization into cells, as it is known that guanidinium-rich dendritic polymers show higher cellular uptake compared to the corresponding amino-rich polymers, with the higher generation dendritic polymers more efficiently internalized into cells [37,38,39,41,62,67]. Hence, the cytotoxicity of GPEI-functionalized oxCNTs can be attributed to a combination of the cytotoxicities of both components, oxCNTs and GPEIs, and for this reason, oxCNTs@GPEI25K exhibits higher toxicity than oxCNTs@GPEI5K due to its higher polymeric content (27.5% in oxCNTs@GPEI25K instead of 22.7% in oxCNTs@GPEI5K), which is found to be more toxic than GPEI5K (Figure S7).

### 3.5. In Vitro Cellular Uptake of DOX-Loaded GPEI-Functionalized oxCNTs

**Figure 9:**
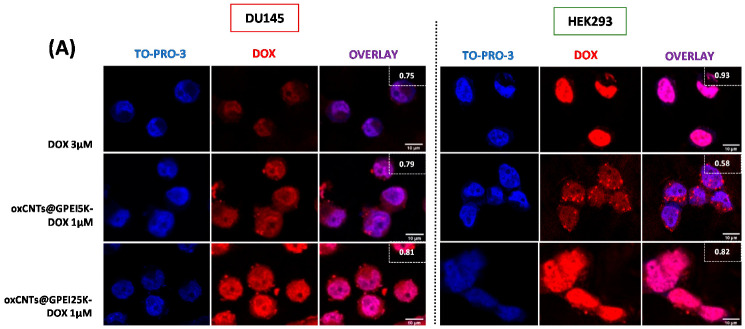

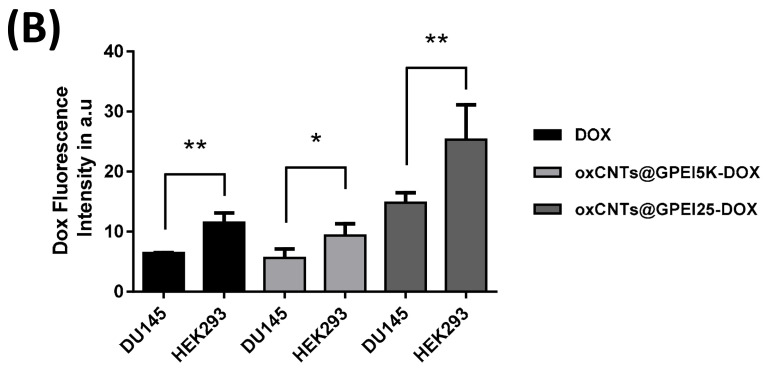
Comparison of DOX internalization in DU145 and HEK293 cells. Cells were incubated with DOX-loaded oxCNTs@GPEI5K or oxCNTs@GPEI25K nanocarriers (1 μΜ DOX concentration) or free DOX (3 μΜ) for 3 h. (**A**) Confocal images depicting DOX cell internalization and Pearson’s co-localization coefficient (in overlay). (**B**) % Quantification of total dox fluorescence between cell lines. Scale bar 10μm. TO-PRO-3 is a nucleus marker (pseudo color blue). Significance was calculated with the Student *t*-test. * *p* > 0.05, ** *p* > 0.01.

**Figure 10:**
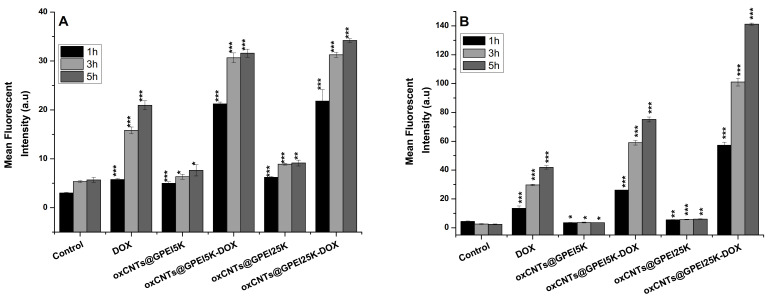
DOX cell uptake in DU145 (**A**) and HEK293 (**B**) cells, measured by flow cytometry. Cells were treated with free DOX and DOX loaded on oxCNTs@GPEI5K or oxCNTs@GPEI25K nanocarriers as well as with unloaded nanocarriers for 1, 3 and 5 h. In all experiments, the DOX concentration was 1 μM, and the corresponding nanocarriers’ concentration was 2 μg/mL. Significance was calculated with the Student *t*-test. * *p* > 0.05, ** *p* > 0.01, *** *p* > 0.001.

To study the internalization properties of free and loaded DOX to GPEI-functionalized oxCNTs, DU145 cells were selected and compared to normal HEK293 cells. Initially, confocal microscopy was used to measure the total DOX fluorescence, as well as co-localization analysis, calculated with Pearson’s correlation coefficient (PCC), applied to detect compartmentalization of internalized free and loaded DOX. In all cases, a low DOX concentration was applied in order to avoid significant cell death during the incubation period. Thus, for these experiments, a low concentration of loaded DOX (1 μΜ) was selected to compare the internalization of oxCNTs@GPEI5K-DOX and oxCNTs@GPEI25K-DOX systems, while a high concentration of free DOX (3 μΜ) was used as positive control, for comparison purposes. After a 3 h treatment, more efficient internalization of DOX was observed, regardless of its status (free or loaded), in HEK293 cells compared to DU145 (Figure 9A). This is further confirmed with flow cytometry experiments, where 1, 3 and 5 h treatment resulted in a greater time-dependent internalization of both free and loaded DOX (1 μΜ) in HEK293 cells, compared to DU145 cells (Figure 10). Specifically, after a 3 h incubation period, the fluorescence intensity of HEK293 cells treated with free DOX or oxCNTs@GPEI5K-DOX was two times that of DU145 cells, while the fluorescence intensity of HEK293 cells treated with oxCNTs@GPEI25K-DOX was ~3-fold that of DU145 cells, suggesting a more efficient cellular uptake in HEK293 cells than that of DU145. Although the drug uptake of loaded DOX was higher in normal cells compared to cancer cells, under the same conditions, greater internalization does not necessarily translate to higher toxicity, as DU145 cells were more sensitive to loaded DOX than HEK293 cells, mainly in the case of oxCNTs@GPEI5K-DOX (Figure 8A,C). In contrast, free DOX internalization in HEK293 cells was accompanied by higher toxicity, compared to DU145 cells. This means that the profiles of DOX uptake are not fully equivalent to the DOX anticancer activity, possibly due to different compartmentalization. Indeed, as observed in Figure 9, in the case of HEK293 cells, free DOX was entirely located in the nucleus (PCC 0.93), whereas the oxCNTs@GPEI5K-DOX system was located both in the nucleus and the cytoplasm (PCC 0.58). On the contrary, in DU145 cells, the oxCNTs@GPEI5K-DOX system was located mainly in the nucleus (PCC 0.78) as in the case of free DOX (PCC 0.75). Thus, we assume that due to a lack of multidrug resistance receptors in normal cells, free DOX is rapidly accumulated in the cell nucleus, whereas loaded DOX is retained in cytoplasmic organelles such as lysosomes and endosomes with delayed release, as other studies suggest [88]. On the other hand, cancer cells are able to efflux free DOX, inducing drug resistance and therefore reducing toxicity. The cellular uptake results are consistent with existing literature where rapid intercalation of DOX is observed with nuclear DNA, delivered into the cells mainly through diffusion [92], whereas the major cell delivery mechanism of nanoparticles is endocytosis, with decreased nuclear localization [93]. In detail, the well-dispersed carbon nanotubes are efficiently internalized by cells though endocytosis or direct membrane penetration and localized in the cytosol [94]. This observation is in agreement with our results, where both GPEI-functionalized oxCNTs are efficiently internalized by cells and located in the cytoplasm around the nucleus (Figure S7). It should also be noted that the presence of guanidinium groups plays a crucial role on the cellular internalization process since it is known that the guanidinium-rich polymers exhibit high cellular uptake, especially compared to the amino-rich analogues, due to the higher binding with cellular membranes. This is achieved due to the strong interaction of guanidinium groups with the anionic, bidentate hydrogen bond acceptor groups of the cellular membrane’s components (carboxylate, phosphate, or sulfate groups) [35,38,39,67]. Moreover, it was found that the cellular uptake of oxCNTs@GPEI25K was higher than that of oxCNTs@GPEI5K due to the higher polymeric content in oxCNTs@GPEI25K as well as to the higher molecular weight of GPEI25K, which enhanced its cell-penetrating capability. As mentioned before, the cellular uptake of guanidinylated dendritic polymers with high molecular weights is more effective than that of low molecular weight analogues [37,41]. For this reason, loaded DOX can be efficiently internalized into cells together with functionalized oxCNTs and then released from the cytosol to the nucleus. Clearly, in cancer cells, DOX is released to the nucleus from the oxCNTs@GPEI5K nanocarrier, localized in the cytosol (Figure S8) in a much faster rate, resulting in greater toxicity compared to normal cells, thus increasing DOX efficiency. This “selectivity” may be associated with the higher lysosomal fragility of the cancer cells, compared to normal cells [95]. In more detail, a recent study in various types of cancer cells has shown that mixed-charge nanoparticles, containing positively and negatively charged groups, similar to oxCNTs@GPEIs nanocarriers, accumulated as large aggregates into cancer lysosomes after a series of distinct pH-dependent aggregation events, from which they cannot be cleared through exocytosis, resulting in increased osmotic pressure inside the lysosomes, forcing them to swell. The swelling increased the permeability of the lysosomal membrane, which eventually resulted in cell death. Instead, the studied nanoparticles were cleared by exocytosis, causing no harm to normal cells [96]. Similar phenomena may occur in our case, where the oxCNTs@GPEI5K-DOX system cannot escape from lysosomes of cancer cells leading to their swelling and finally to cell death, unlike in normal cells. Furthermore, as the environment in a solid tumor is generally more acidic (pH = 6.5–6.9) than that of a normal tissue (pH ≈ 7.4), and as lysosomes have an even more acidic lumen (pH ≈ 4.8) [85], other studies [58,59] and our own data suggest that DOX is more rapidly released from oxCNTs@GPEI5K carrier under mild acidic environment than physiological pH, exerting its toxicity faster due to lysosomal disruption. On the contrary, the oxCNTs@GPEI25K-DOX system does not exhibit analogous specificity with that of oxCNTs@GPEI5K-DOX since it is internalized fast in both cancer and normal cells, and DOX is rapidly released from the oxCNTs@GPEI25K nanocarrier, located in the cytosol (Figure S8). Finally, the released DOX is localized almost entirely in the cell nucleus (PCC 0.81 in DU145 and PCC 0.82 in HEK293), resulting in high toxicity regardless of the cell’s status (Figure 8). This different behavior may be attributed to the enhanced cellular uptake of oxCNTs@GPEI25K compared to that of oxCNTs@GPEI5K (Figure S8) as well as to the significantly faster release rate of DOX from oxCNTs@GPEI25K than that of oxCNTs@GPEI5K under both tested pH environments (Figure 7). Therefore, based on our results, it can be suggested that the oxCNTs@GPEI5K-DOX system can be used as an efficient platform for selective action of DOX to cancer cells.

### 3.6. Effects of DOX-Loaded GPEI-Functionalized oxCNTs on Apoptosis/Necrosis in DU145 Cells

**Figure 11:**
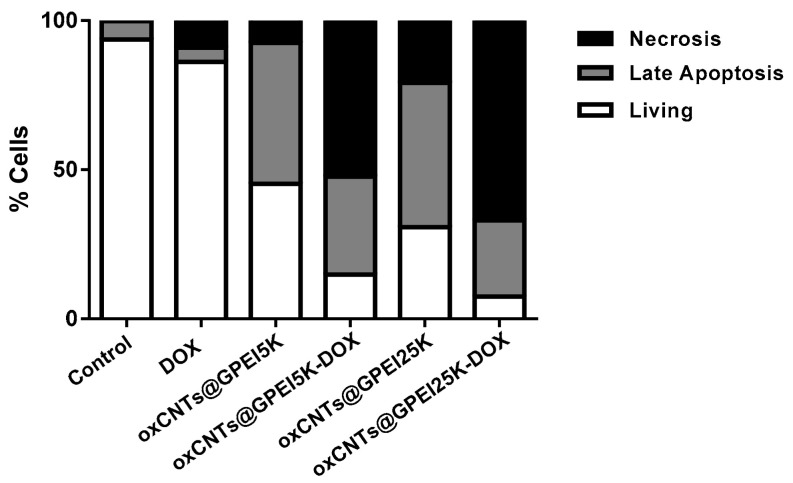
Quantification of living, late apoptotic and necrotic percentage of DU145 cells treated with free DOX and DOX loaded on oxCNTs@GPEI5K or oxCNTs@GPEI25K nanocarriers as well as with unloaded nanocarriers for 3 h, measured with Annexin V-FITC/7-AAD staining. In all experiments, the DOX concentration was 1 μM, and the corresponding nanocarriers’ concentration was 2 μg/mL.

The apoptosis/necrosis of DU145 cells was studied in order to elucidate the mechanism of anticancer activity of DOX-loaded GPEI-functionalized oxCNTs. Specifically, the quantification of living, late apoptotic and necrotic percentage of DU145 cells treated with free DOX and DOX loaded on oxCNTs@GPEI5K or oxCNTs@GPEI25K nanocarriers (DOX concentration was 1 μM) as well as with unloaded nanocarriers for 3 h was performed using Annexin V-FITC/7-AAD double staining and measured by flow cytometry. It is known that Annexin V-FITC labels apoptotic cells with exposed phosphatidylserines (PS), while 7-AAD stains necrotic cells with membrane damage [97]. As shown in Figure 11, 3 h treatment with the oxCNTs@GPEI25K carrier promotes higher late cell apoptosis and necrosis compared to oxCNTs@GPEI5K, suggesting greater disruption of the cell membrane integrity, probably due to its higher polymeric content, thus resulting in higher internalization into cells and eventually in higher toxicity. Moreover, cell treatment with DOX loaded on both nanocarrier systems resulted in much higher percentage of both late apoptotic and necrotic cells compared to treatment with the same concentration of free DOX, with the oxCNTs@GPEI25K-DOX system exhibiting the highest percentage, which justifies the highest DOX anticancer activity. Absence of early apoptotic cells indicates a more abrupt and uncontrolled cell death mechanism attributed to the faster endocytosis and intracellular release of DOX from the nanocarriers. Therefore, both DOX-loaded systems are able to induce cell death by apoptosis and mainly by necrosis, with the oxCNTs@GPEI25K-DOX system being more toxic to cells compared to the oxCNTs@GPEI5K-DOX system.

## 4. Conclusions

An efficient drug delivery system for doxorubicin with specific toxicity against tumor cells was prepared, based on oxidized multi-walled carbon nanotubes (oxCNTs) functionalized with guanidinylated dendritic molecular transporters through non-covalent interactions. Thus, guanidinylated derivatives of hyperbranched polyethyleneimine with molecular weight 5000 and 25,000 Da (GPEI5K and GPEI25K), having analogous chemical structure with known molecular transporters such as the guanidinylated poly(propylene imine) dendrimers, were synthesized in order to be used as transporting agents for drug delivery. Subsequently, these guanidinylated PEI derivatives interacted electrostatically and also through hydrogen bonding and van der Waals attraction forces with oxCNTs, affording hybrid materials (oxCNTs@GPEI5K and oxCNTs@GPEI25K) with GPEI loading ranging approximately between 22% and 27%. The obtained functionalized CNTs were structurally characterized using various techniques, such as FTIR, Raman, XPS, NMR, etc., revealing the successful attachment of GPEIs on the surface of oxCNTs, while the homogenous wrapping of GPEIs all over the sidewalls of oxCNTs was microscopically confirmed by SEM and TEM. Moreover, as revealed by visual observation over time, UV–vis spectroscopy and ζ-potential measurements, these hybrids can efficiently disperse in aqueous media, affording stable aqueous dispersions for at least 6 months. These enhanced dispersion properties can be attributed to the presence of guanidinium groups on the surface of the oxCNTs that not only induces hydrophilicity resulting in high aqueous compatibility, but also provides the surface charge necessary to cause electrostatic repulsion inhibiting their agglomeration. Then, doxorubicin (DOX), a widely used anticancer drug, was efficiently loaded on the prepared hybrid materials to yield DOX-loaded systems. These systems exhibited high DOX loading and encapsulation efficiency due to not only the π–π stacking interactions between the DOX and the CNTs, but also the ability of dendritic polymers to efficiently encapsulate doxorubicin. DOX-loaded oxCNTs@GPEIs systems exhibited pH-triggered release. It was found that DOX can be efficiently released from both systems in acidic environments, with faster release rate from oxCNTs@GPEI25K. In vitro experiments in cancer and normal cells demonstrated a rapid internalization of loaded DOX on the oxCNTs@GPEI5K nanocarrier system, with specific toxicity against cancer in contrast to normal cells. Additionally, it significantly increased DOX efficiency compared to the free drug, resulting in high toxicity in a non-apoptotic, fast and catastrophic manner that cancer cells cannot recover from. Even though loaded DOX was found to be more efficiently internalized and released from the oxCNTs@GPEI25K compared to the oxCNTs@GPEI5K system in the experimental conditions tested herein, this system failed to show specificity against cancer cells, perhaps suggesting it should be administered in even lower concentrations for biological experiments. Overall, we conclude that the oxCNTs@GPEI5K nanocarrier is a potent and efficient nanoscale DOX delivery system that exhibits significant selective toxicity against cancerous cells, constituting a promising candidate for cancer therapy. Moreover, the unique combination of multi-walled carbon nanotubes and dendritic molecular transporters to prepare nanocarriers with enhanced cell-penetration capability can be applied as an effective strategy to develop different drug delivery systems for cancer-specific delivery of various anticancer drugs, able to be used for various cancer therapy applications.

## Data Availability

Not Applicable.

## Supplementary Materials

The following are available online at https://www.mdpi.com/article/10.3390/pharmaceutics13060858/s1, Figure S1: ^1^H NMR spectra (500 MHz, D_2_O) of GPEI5K (upper part) and GPEI25K (lower part). Figure S2: ^13^C NMR spectra (125.1 MHz, D_2_O) of GPEI5K (upper part) and GPEI25K (lower part). Figure S3: XPS survey spectra of oxCNTs@GPEI5K and oxCNTs@GPEI25K. Figure S4: XPS high resolution spectra for the C1s (a,b) and N1s (c,d) of GPEI5K and GPEI25K. Figure S5: XPS high resolution for the C1s of oxCNTs. Figure S6. ^1^H NMR spectra (500 MHz, D_2_O) of oxCNTs@GPEI5K (upper part) and oxCNTs@GPEI25K (lower part), including maleic acid as internal standard. Figure S7. Comparative toxicities of oxCNTs, GPEI derivatives and the corresponded parent polymers (PEI5K and PEI25K) on carcinoma DU145 and normal HEK293 cells following incubation at various concentrations for 3h as determined by MTT assays 24h following incubation. Significance of GPEIs was calculated with the student t-test compared to their respective parent polymers. * *p* > 0.05, ** *p* > 0.01, *** *p* > 0.001, while no annotation implies no statistical significance, *p* > 0.05. Figure S8: Confocal images of DU145 cells treated with 2μg/mL rhodamine-labelled oxCNTs@GPEI5K (A,B) and oxCNTs@GPEI25K (C,D) for 3h. Table S1: Atomic percentage % of GPEI-functionalized oxCNTs. Table S2: Elemental analysis results of oxCNTs, GPEI and GPEI-functionalized oxCNTs.
