# Supplementary material for: Multi-Walled Carbon Nanotubes Decorated with Guanidinylated Dendritic Molecular Transporters: An Efficient Platform for the Selective Anticancer Activity of Doxorubicin
Source: Pharmaceutics. 2021 Jun 9;13(6):858. doi: 10.3390/pharmaceutics13060858 (PMC8226981; doi:10.3390/pharmaceutics13060858)
Supplement: Supplementary file 1 [file pharmaceutics-13-00858-s001.zip › pharmaceutics-1214224-supplementary.pdf]

pharmaceutics

MDPI

Supplementary Materials: Multi-Walled Carbon Nanotubes Decorated with Guanidinylated Dendritic Molecular Transporters: An Efficient Platform for the Selective Anticancer Activity of Doxorubicin

Kyriaki-Marina Lyra, Archontia Kaminari, Katerina N. Panagiotaki, Konstantinos Spyrou, Sergios Papageorgiou, Elias Sakellis, Fotios K. Katsaros and Zili Sideratou

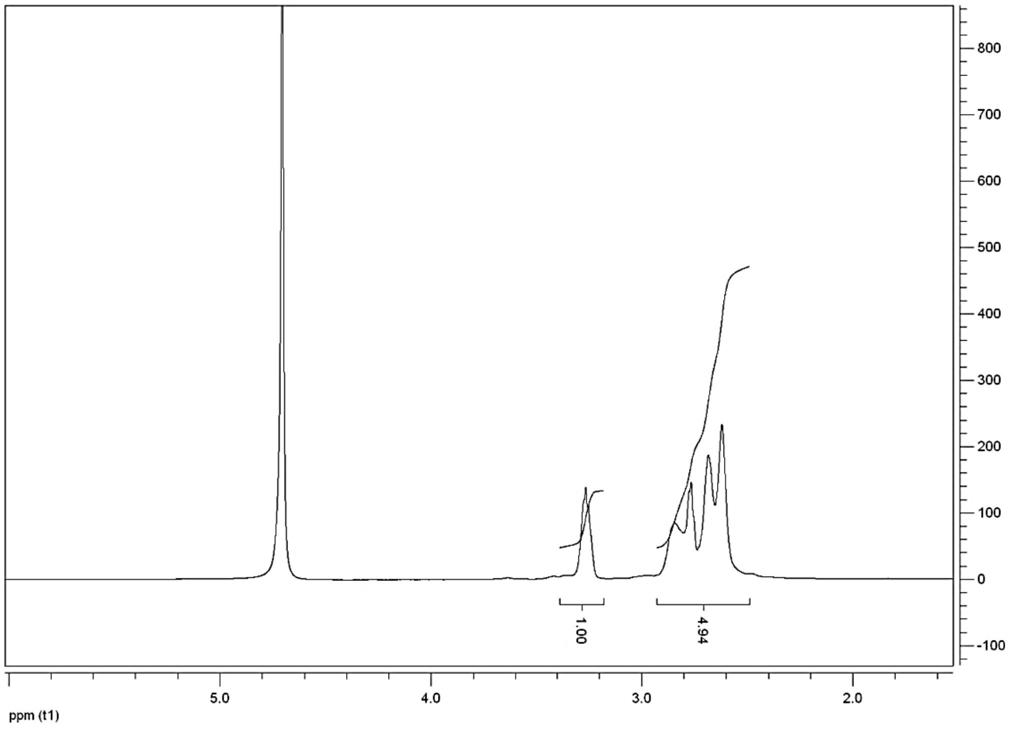

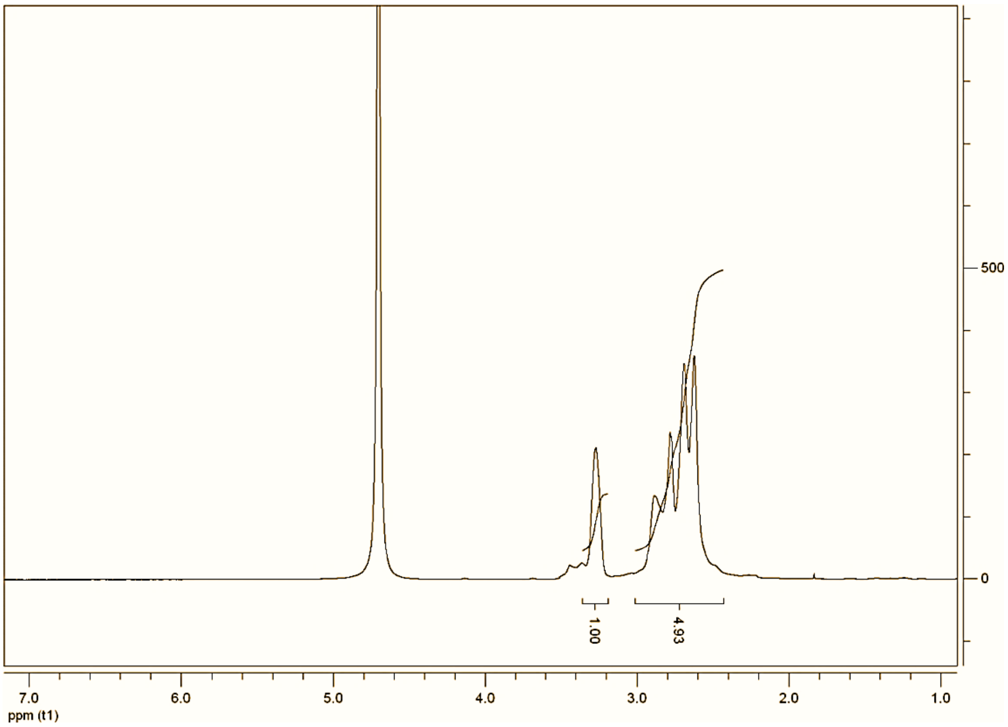

Figure S1. ^{1}H NMR spectra (500 MHz, D_2O) of GPEI5K (upper part) and GPEI25K (lower part).

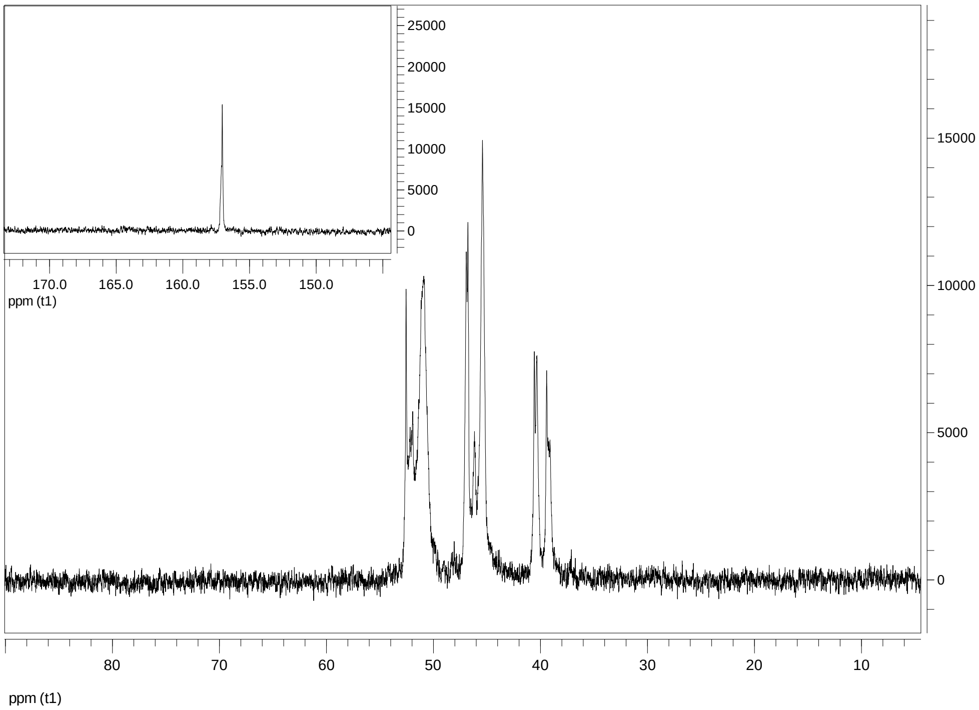

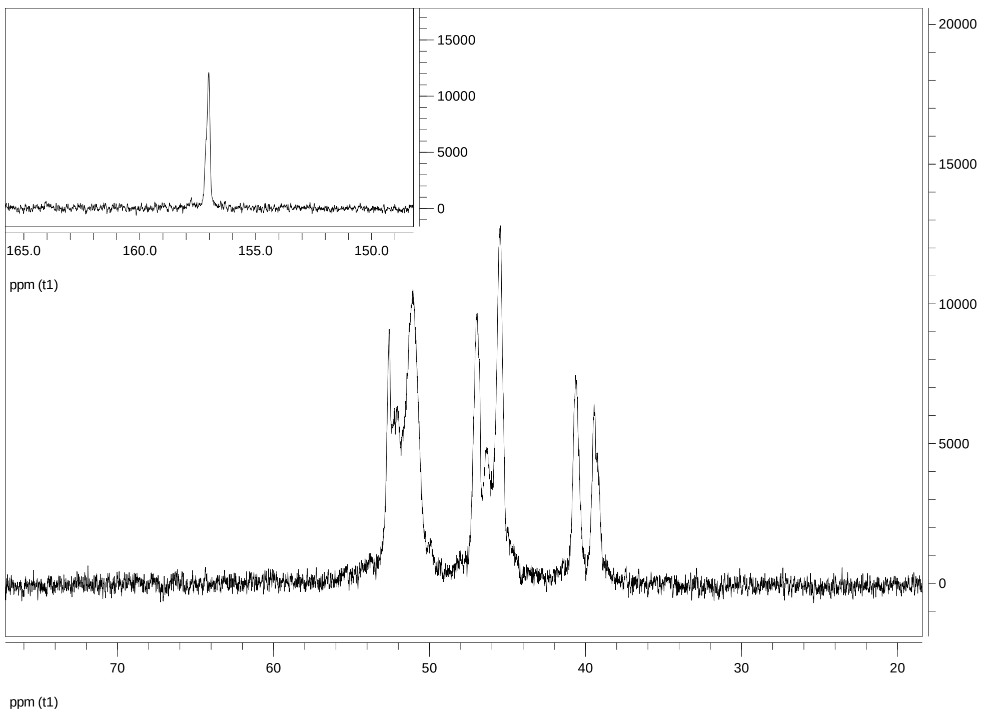

Figure S2. ^{13}C NMR spectra (125.1 MHz, D_2O) of GPEI5K (upper part) and GPEI25K (lower part).

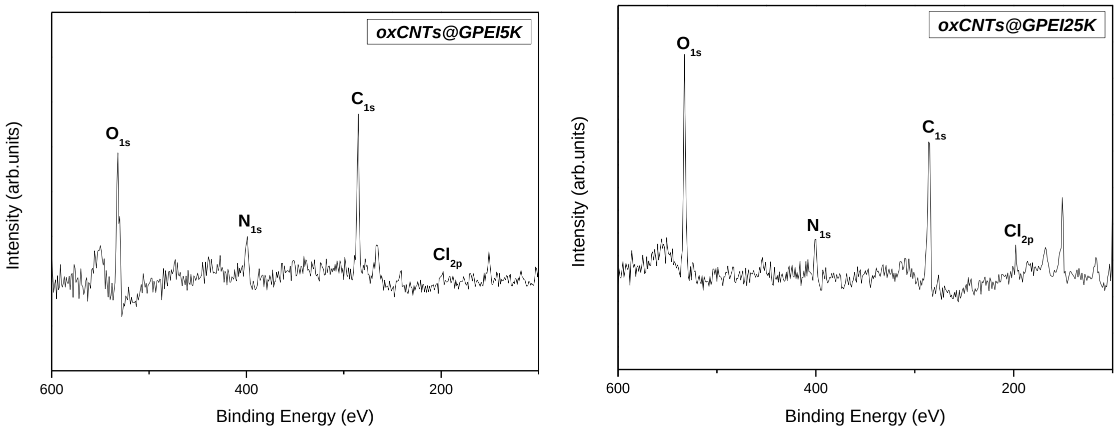

Figure S3. XPS survey spectra of oxCNTs@GPEI5K and oxCNTs@GPEI25K.

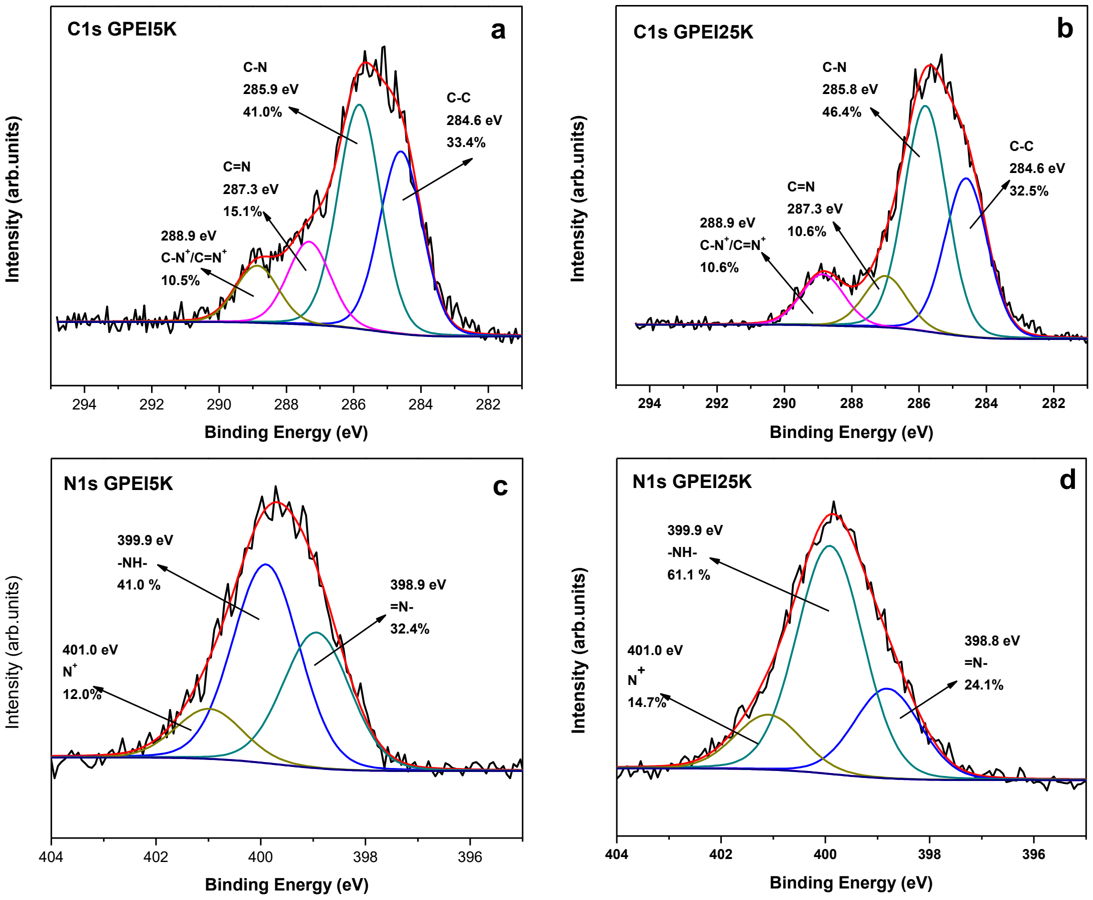

Figure S4. XPS high resolution spectra for the C 1s (**a, b)** and N 1s (**c, d**) of GPEI 5K and GPEI 25K.

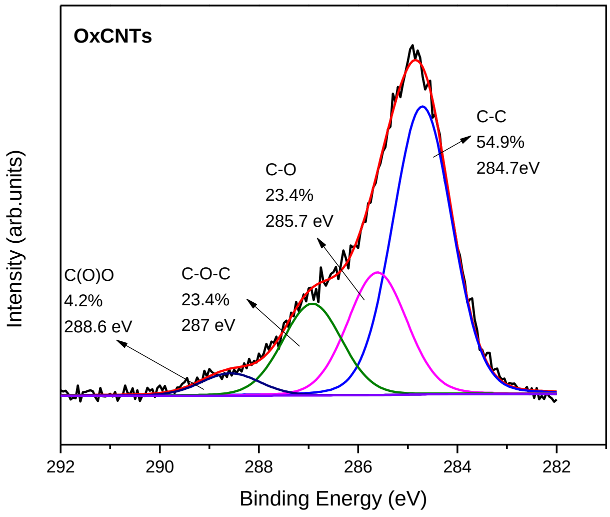

Figure S5. XPS high resolution for the C1s of oxCNTs.

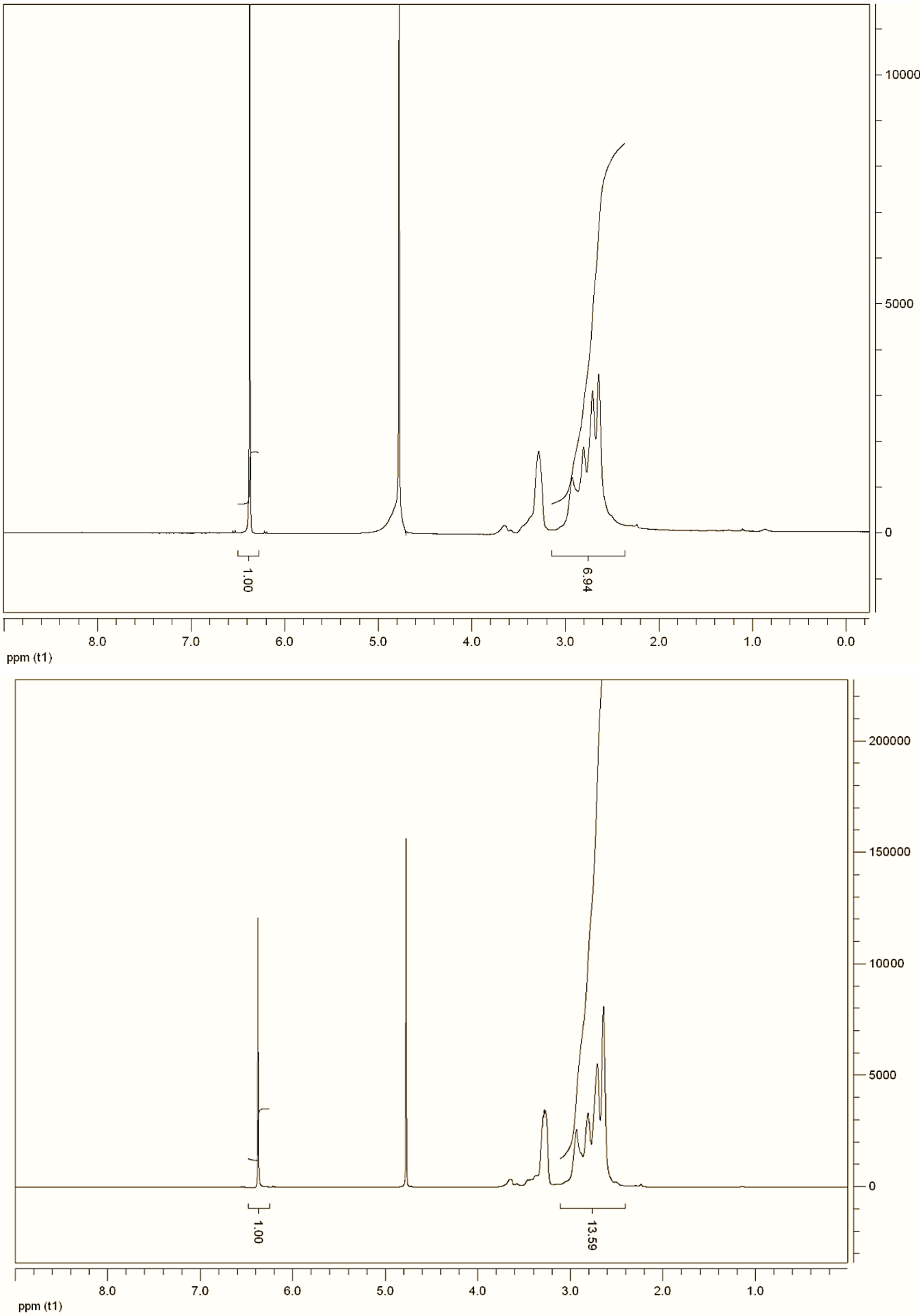

Figure S6. ^{1}H NMR spectra (500 MHz, D_2O) of oxCNTs@GPEI5K (upper part) and oxCNTs@GPEI25K (lower part), including maleic acid as internal standard.

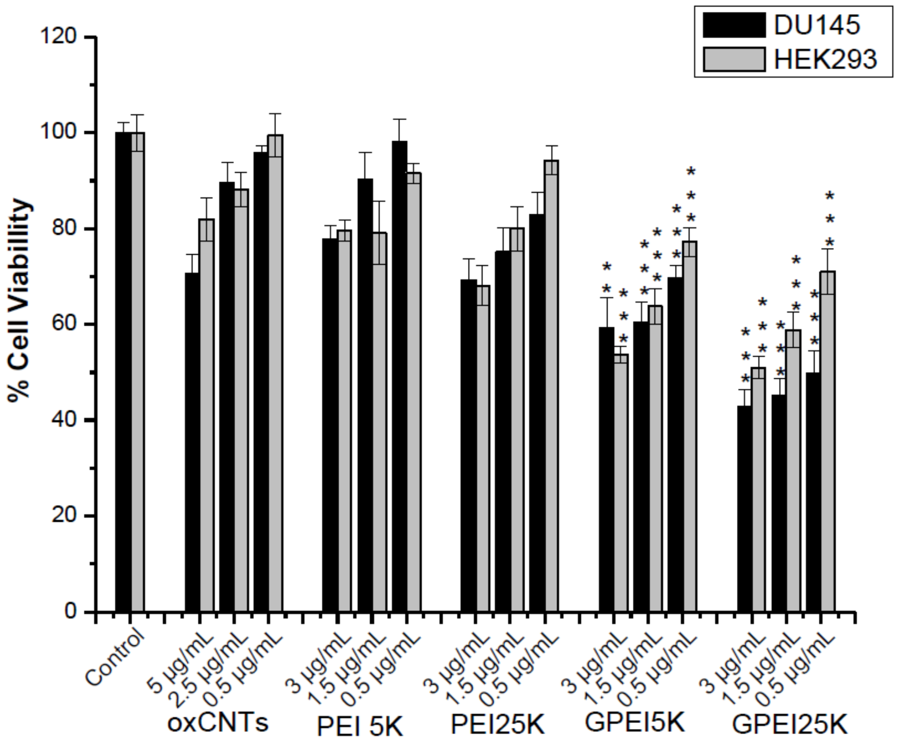

Figure S7. Comparative toxicities of oxCNTs, GPEI derivatives and the corresponded parent polymers (PEI5K and PEI25K) on carcinoma DU145 and normal HEK293 cells following incubation at various concentrations for 3h as determined by MTT assays 24h following incubation. Significance of GPEIs was calculated with the student t-test compared to their respective parent polymers. **p* > 0.05, ***p* > 0.01, ****p* > 0.001, while no annotation implies no statistical significance, *p* > 0.05.

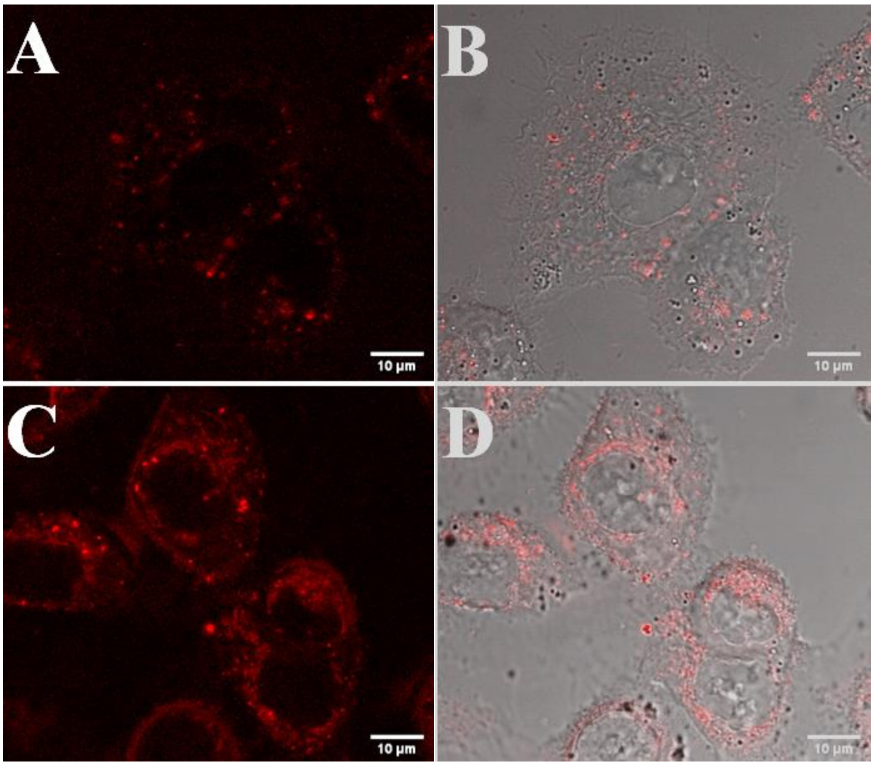

Figure S8. Confocal images of DU145 cells treated with 2μg/mL rhodamine-labeled oxCNTs@GPEI5K **(A,B)** and oxCNTs@GPEI25K (**C**,**D**) for 3h.

Table S1. Atomic percentage % of GPEI-functionalized oxCNTs.

Element	oxCNTs@GPEI 5K	oxCNTs@GPEI 25K
	% Atomic Percentage	
Carbon	62.8	67.3
Oxygen	33.2	24.3
Nitrogen	4	8.4
N/C ratio	0.06	0.12

Table S2. Elemental analysis results of oxCNTs, GPEI and GPEI-functionalized oxCNTs.

Sample	Sample elemental composition/wt%			GPEI (% w/w)
	C	H	N	
oxCNTs	91.52	1.86	0.23	
GPEI5K	35.63	10.26	32.32	
oxCNTs@GPEI5K	69.54	2.08	7.51	22.7%
GPEI25K	38.19	8.52	33.56	
oxCNTs@GPEI25K	59.29	1.51	9.40	27.5 %
